# Manipulating Surface Chemistry on the Microarchitecture of Coal‐Based Hard Carbon for Improved Sodium Storage

**DOI:** 10.1002/advs.202513835

**Published:** 2025-09-23

**Authors:** Wenhai Zhang, Ruizhen Song, Hong Meng, Yakun Tang, Yue Zhang, Lang Liu, Ping Han, Limin Deng, Yuliang Cao

**Affiliations:** ^1^ State Key Laboratory of Chemistry and Utilization of Carbon Based Energy Resources College of Chemistry Xinjiang University Urumqi Xinjiang 830017 P. R. China; ^2^ Engineering Research Center of Organosilicon Compounds & Materials of Ministry of Education College of Chemistry and Molecular Sciences Wuhan University Wuhan 430072 P. R. China

**Keywords:** coal, hard carbon, low‐cost, oxygen‐containing functional groups, sodium‐ion batteries

## Abstract

The aromatic nature of coal results in highly graphitized hard carbon (HC), which significantly impacts its sodium storage performance. Constructing oxygen‐containing functional groups (OFGs) can effectively enhance sodium storage performance, but the mechanistic role of OFGs in governing the surface chemical evolution of coal‐based HC remains poorly understood. Herein, OFGs are introduced into coal molecules through various pre‐oxidation methods. Comprehensive in situ/ex situ testing elucidated that different OFGs have different effects on the intramolecular rearrangement of coal. Compared with C═O, ‐OH, and C─O─C groups, the carboxyl can inhibit decarboxylation during pyrolysis, raising the upper limit of the temperature window for intramolecular carbon rearrangement from 500 to 600 °C. This effect reduces intermolecular condensation efficiency during carbonization, thereby suppressing soft carbon formation. The strategy concurrently enlarges graphite‐like interlayer spacing and creates closed pores, ultimately enhancing the sodium storage capacity of coal‐based HC. The optimized HC shows enhanced capacity (308 mAh g^−1^) with a 1.4 times increase in low‐voltage plateau capacity compared to the unmodified HC. This work elucidates the structure‐function relationship between specific OFGs and carbonization behavior, develops a practical strategy to modulate coal's molecular rearrangement via targeted surface chemistry, and contributes to achieving low‐cost, high‐performance HC in advanced SIBs.

## Graphical abstract

This study utilizes surface chemistry engineering to enhance coal‐based carbon materials for sodium storage. By introducing oxygen‐containing functional groups (OFGs) via pre‐oxidation, the researchers found that carboxyl groups delay decarboxylation during pyrolysis, enabling extended intermolecular rearrangement up to 600 °C while suppressing soft carbon formation. The modified structure exhibits enlarged graphene interlayer spacing and generates closed pores, significantly improving sodium storage capacity in hard carbon.

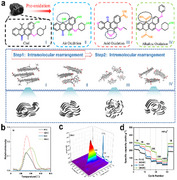

## 1 Introduction

Sodium‐ion batteries (SIBs) present a promising alternative for large‐scale production, primarily due to the abundance and low cost of sodium resources.^[^ ^1^ ^]^ They also offer safety advantages and compatibility with existing lithium‐ion battery (LIBs) production facilities.^[^ ^2^ ^]^ Despite these benefits, the development of low‐cost, high‐performance anode materials remains a significant challenge, hindering the further advancement of SIBs.^[^ ^3^ ^]^ Recent studies have explored various materials, including metal oxides^[^ ^4^ ^]^ phosphates,^[^ ^5^ ^]^ organic materials,^[^ ^6^ ^]^ and carbon materials^[^ ^7^ ^]^ as potential anodes for SIBs. Among these, hard carbon (HC) has garnered considerable attention, which is regarded as the most promising anode material for commercialization because of its high specific capacity, excellent cycling stability, and the wide availability of its raw materials.^[^ ^8^, ^9^ ^]^ Nevertheless, some severe issues still remained, hindering the industrialization process, particularly achieving a balance between cost and performance.^[^ ^10^ ^]^

The choice of precursors is crucial in addressing the aforementioned issue. Currently, the precursors employed for the production of HC are mainly classified into three distinct categories: polymer compounds,^[^ ^11^, ^12^ ^]^ biomass,^[^ ^13^, ^14^ ^]^ and fossil fuels.^[^ ^15^, ^16^ ^]^Although biomass resources are abundant, renewable, and have high capacity, they are subject to seasonal variations and have low carbon residue rates (averaging ≈10%).^[^ ^17^ ^]^ Polymer precursors allow precise regulation of the microstructure, including pore structure and active sites, at the molecular level. Despite this advantage, they are expensive and difficult to industrialize.^[^ ^18^ ^]^ Coal, as a fossil fuel, is regarded as a promising precursor for HC due to its extensive global distribution, substantial reserves, cost‐effectiveness, and high carbon residue rate, primarily consisting of cross‐linked polycyclic aromatic hydrocarbon (PAH) frameworks embedded with inorganic minerals and free organic molecules (such as light hydrocarbons and compounds containing oxygen/nitrogen functional groups).^[^ ^19^, ^20^ ^]^ Its pyrolysis process mainly includes the following stages: drying and degassing stage (≈350 °C), melting and rearrangement stage (350–500 °C), solidification stage (500–650 °C), and polycondensation to coke stage (>650 °C).^[^ ^21^ ^]^ The regulation of the molten rearrangement stage is crucial for the construction of high‐performance sodium storage HC.^[^ ^22^ ^]^ During this process, coal molecules first undergo intramolecular rearrangement driven by dehydrogenation or deoxygenation, forming stabilized cyclic structures. Subsequently, the rearranged cyclic coal molecules further undergo intermolecular rearrangement. Given the limited number of cross‐linkable functional groups within coal molecules, the intermolecular rearrangement proceeds rapidly, facilitating extensive intermolecular fusion and rearrangement. This transformation pathway ultimately yields HC with an enhanced graphitization degree. The surface‐controlled chemical process of modulating intramolecular rearrangement in coal to alter pyrolytic carbon structures remains insufficiently investigated and systematically elucidated.

Numerous researchers have explored some oxidation methods, such as air oxidation and acid oxidation, to alter the molecular structure of coal and its derivatives, thereby inhibiting the graphitization process.^[^ ^23^ ^]^ For example, Wu et al. employed air pre‐oxidation to pretreat coal, yielding a HC anode with a notably high plateau capacity.^[^ ^24^ ^]^ Zhao and co‐workers pretreated coal using acid pre‐oxidation, and the optimal HC exhibited an improved reversible capacity of 259.7 mAh g^−1^ after 50 cycles at 0.03 A g^−1^.^[^ ^25^ ^]^ Although the pre‐oxidation process effectively enhances the sodium storage performance of coal‐based HC, the mechanism underlying the construction of coal's surface chemical processes remains unclear, and the specific roles and behaviors of surface oxygen‐containing functional groups (OFGs) require further investigation. The oxidation methods previously proposed typically introduce OFGs such as hydroxyl (‐OH), ether (C‐O‐C), carboxyl, and ester groups into coal molecules, but their precise roles in inhibiting intramolecular rearrangement and controlling carbonization structures need detailed examination.

In this study, by employing a simple alkali‐oxygen oxidation method, we found that the types of OFGs obtained are completely different from those produced by traditional air oxidation and acid oxidation. The alkali‐oxygen oxidation method primarily generates carboxyl functional groups. Based on in situ thermogravimetric‐infrared‐mass spectrometry (TG‐IR‐MS) analysis, it is observed that compared to other functional groups, the carboxyl groups incorporated through alkali‐oxygen oxidation extend the intramolecular rearrangement window of coal from 200–500 °C to 200–600 °C, effectively inhibiting intermolecular reorganization, thereby suppressing intramolecular rearrangement and preventing the development of soft carbon structures. The proposed alkali‐oxygen oxidation method not only facilitates the targeted introduction of carboxyl groups but also effectively eliminates ash and small organic molecules from the coal matrix. This results in an expansion of the interlayer spacing and an increase in the closed pore volume of the coal‐derived HC. The optimized coal‐based HC demonstrates an enhanced capacity of 308 mAh g^−1^ with initial coulombic efficiency (ICE) up to 80.1%, with a 1.4‐times increase in low‐voltage plateau capacity over the unmodified HC (212 mAh g^−1^). This work focuses on the surface chemistry of coal‐based HC and the manipulation of OFGs to achieve inhibition of intramolecular rearrangement during pyrolysis. This approach aims to obtain an HC microstructure more favorable for sodium storage, thereby enhancing its sodium storage performance and practical application.

## 2 Results and Discussion

**Figure 1:**
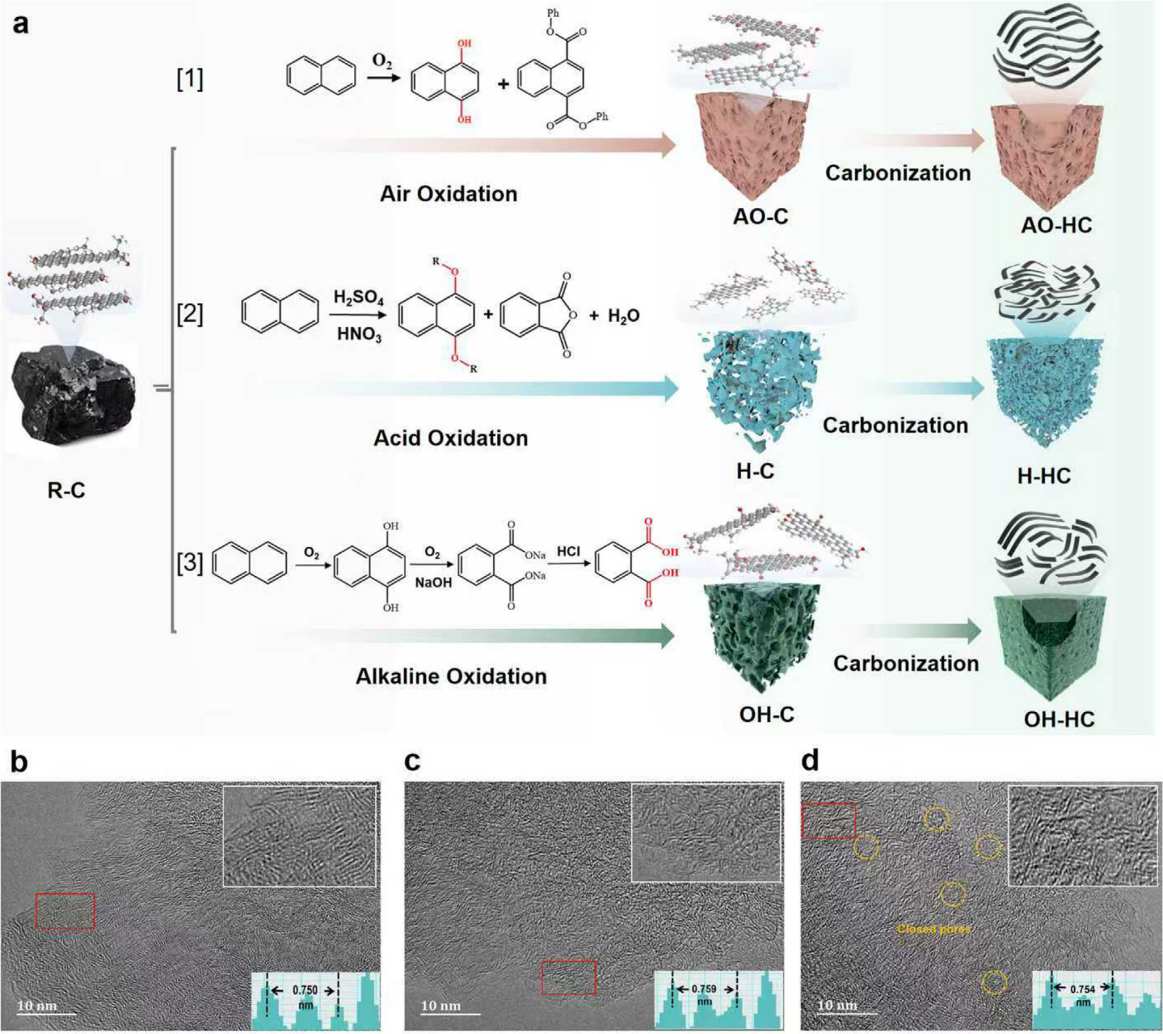
a) Schematic illustration of the synthesis process and its corresponding HRTEM images of AO‐HC b), H‐HC c), and OH‐HC (d).

**Figure 2:**
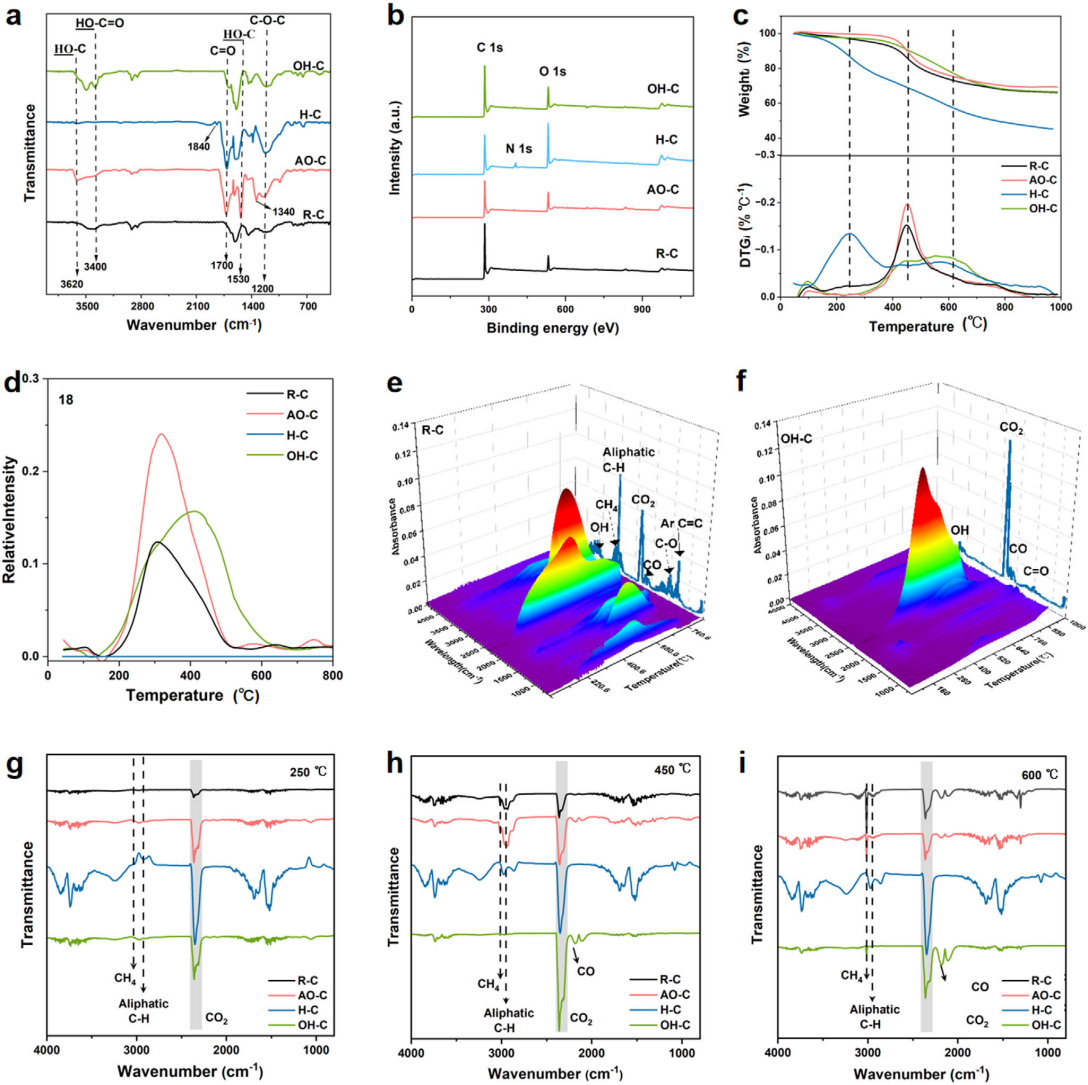
FTIR spectra a), XPS survey spectra b), TG‐DTG curves c), and in situ normalized mass spectra of H_2_O evolution during pyrolysis d) of R‐C, AO‐C, H‐C, and OH‐C. The 3D TG‐FTIR spectra of R‐C e) and OH‐C (f). The FTIR spectra of pyrolysis gases from different precursors at 250 °C j), 450 °C h), and 600 °C (i).

**Figure** **1**a illustrates the synthesis routes of AO‐HC, H‐HC, and OH‐HC hard carbons, prepared via air oxidation, mixed acid oxidation, and alkali‐oxygen oxidation pretreatment, respectively (The specific experimental process can be found in the supporting information). The corresponding high‐resolution transmission electron microscopy (HRTEM) images reveal that different pre‐oxidation methods significantly affect the microcrystalline structure of HC, including the interlayer spacing of graphite and the dimensions of microcrystals (Figure 1b–d). The specific reasons for these effects will be analyzed in detail later. Fourier transform infrared (FTIR) spectra analysis was conducted to characterize the surface OFGs of R‐C, AO‐C, H‐C, and OH‐C coal‐precursors, prepared by raw, air oxidation, mixed acid oxidation, and alkali‐oxygen oxidation (**Figure** **2**a). The types and contents of OFGs in coal are found to vary significantly after different pre‐oxidation treatments. In the FTIR spectrum of AO‐C obtained through air oxidation, two distinct absorption peaks are observed at 1530 cm^−1^ and 3620 cm^−1^, corresponding to the bending vibration and stretching vibration of ‐OH groups, respectively.^[^ ^23^, ^26^ ^]^ Additionally, two other absorption peaks appeared at 1700 and 1340 cm^−1^, which are attributed to the C═O stretching vibration and C─O stretching vibration of ester groups, respectively.^[^ ^27^ ^]^ Comparative analysis revealed that AO‐C exhibited a higher content of ‐OH groups than other precursors, demonstrating that air pre‐oxidation can effectively introduce ‐OH functional groups. This phenomenon can be explained by the oxidation of short‐chain alkanes in the coal structure during air oxidation, which generates ‐OH groups along with a small quantity of carboxyl groups. These oxygen‐containing functional groups subsequently undergo esterification to form ester groups, as primarily described by Equation 1 in Figure 1.

FTIR analysis of the mixed acid preoxidized coal‐precursor (H‐C) reveals the most intense C─O─C stretching vibration peak at 1200 cm^−1^. Additionally, two characteristic C═O stretching vibrations of anhydride groups are observed at 1700 and 1840 cm^−1^. These findings indicate that under mixed‐acid oxidation conditions, short‐chain alkanes are oxidized to form carboxyl and ‐OH groups, which subsequently undergo decarboxylation and dehydration catalyzed by concentrated sulfuric acid, leading to the formation of anhydride and C─O─C linkages, based on Equation 2 in Figure 1.

In the FTIR spectrum of the OH‐C precursor treated by the alkali‐oxygen oxidation method, in addition to the characteristic peaks of C─O─C and ‐OH stretching vibrations at 1200 and 3620 cm^−1^, distinct absorption bands corresponding to the C═O stretching vibration of carboxyl groups (1700 cm^−1^) and the ‐OH stretching vibration (3400 cm^−1^) were observed. This can be attributed to the oxidation of aromatic molecules and side chains in coal by H_2_O_2_, generating abundant carboxyl and ‐OH groups. Under alkaline conditions, the resulting carboxyl and ‐OH groups were converted into stable ‐COONa and ‐ONa, effectively controlling the oxidation degree of coal molecules by H_2_O_2_.^[^ ^28^ ^]^ Furthermore, the alkaline medium catalyzed both the oxidation and hydrolysis reactions of coal, dissolving free organic molecules within the coal matrix and facilitating further oxidation of its deeper structures by H_2_O_2_.^[^ ^29^ ^]^ Finally, the addition of dilute hydrochloric acid induced substitution and Williamson ether synthesis reactions with ‐COONa and ‐ONa, leading to the formation of carboxyl, ‐OH, and ‐C‐O‐C functional groups.

The chemical composition and elemental states of different precursors were further investigated using X‐ray Photoelectron Spectroscopy (XPS). As shown in Figure 2b, except for the H‐C precursor, the other precursors are composed of C and O elements. The introduction of nitrogen in H‐C is mainly due to nitric acid oxidation. As can be seen from Table S1 (Supporting Information), the oxygen content of the R‐C precursor is only 11.57 at%, while the oxygen contents of the oxidized samples AO‐C, H‐C, and OH‐C are increased to 17.62 at%, 27.82 at%, and 15.27 at%, respectively, indicating that different pre‐oxidation methods can effectively introduce OFGs.

Further analysis of the types and contents of OFGs in the precursors was carried out, and the high‐resolution O 1s spectrum was fitted with four peaks, corresponding to convolution peaks with binding energies of 531.5, 532.6, 533.4, and 534.4 eV, which correspond to C═O, C‐O‐C, ‐OH, and ‐C(O)‐O‐ functional groups (Figure S1a–d, Supporting Information).^[^ ^30^ ^]^ Since the binding energies of carboxyl, ester, and anhydride groups are all ≈534.4 eV, they are represented by the ‐C(O)‐O‐ functional group. Combined with the FTIR spectra, it is known that the functional groups at this position for AO‐C, H‐C, and OH‐C are ester, anhydride, and carboxyl groups, respectively. According to the XPS analysis results (Table S1, Supporting Information), the most abundant OFGs in R‐C, AO‐C, H‐C, and OH‐C are C═O, ‐OH, C‐O‐C, and carboxyl, respectively, among which OH‐C has the highest carboxyl functional group content among all precursors. This result is in good agreement with the FTIR analysis.

Figure 2c presents the experimental and calculated Thermogravimetric (TG) and Derivative Thermogravimetric (DTG) curves for the samples. The weight loss curves of all samples are primarily divided into four stages: drying and degassing stage (≈350 °C), melting and rearrangement stage (350–500 °C), solidification stage (500–650 °C), and polycondensation to coke stage (>650 °C). The TG curves show that the carbon yield of H‐C is only 45%, which is significantly lower than that of the raw coal R‐C (67%). However, the carbon yields of AO‐C and OH‐C are close to that of R‐C, indicating that air pre‐oxidation and alkali‐oxygen oxidation did not destroy the overall structure of the coal, unlike the severe damage caused by mixed acid pre‐oxidation to the coal's molecular structure. R‐C and AO‐C exhibit similar DTG curves, indicating analogous pyrolysis reaction processes. Both samples show weight loss peaks at ≈450 °C, with the peak of AO‐HC slightly shifted to a higher temperature, suggesting that the introduced ‐OH functional groups can moderately inhibit coal pyrolysis.

H‐C displays a distinct weight loss peak at 250 °C, attributed to the decomposition of small molecules generated from the fragmentation of coal macromolecules during mixed acid treatment. Additionally, a weaker weight loss peak is also observable at 250 °C in the DTG curve of R‐C, which is associated with the release of small organic molecules or light hydrocarbons present in the raw coal. In contrast, AO‐C and OH‐C do not exhibit weight loss peaks at these low temperatures. The absence of small organic molecule release in AO‐C may be due to chemical reactions between free oxide molecules and the carbon skeleton during pre‐oxidation or the volatilization of light hydrocarbons. The lack of a weight loss peak in OH‐C can be explained by the effectiveness of the proposed alkali‐oxygen oxidation method in dissolving free organic molecules within the coal matrix, which is consistent with the inferences drawn from the FTIR result.

Compared to other samples, OH‐C exhibits two broad weight loss peaks within the 400–650 °C range, centered at ≈450 and 570 °C, respectively. The weaker peak at 450 °C and the more pronounced peak at the higher temperature (570 °C) suggest that the carboxyl groups introduced via alkali‐oxygen oxidation effectively prolong the relaxation time of coal molecules. As a result, the coal molecules reach their solidification temperature before undergoing sufficient melting and rearrangement, leading to increased structural disorder during pyrolysis. To gain deeper insights into the influence of OFGs on the coal pyrolysis process, we conducted in situ mass spectrometric analysis of the H_2_O released during the pyrolysis of R‐C, AO‐C, H‐C, and OH‐C. As shown in Figure 2d, H‐C exhibits negligible H_2_O release during pyrolysis, indicating that the OFGs in this coal do not produce H_2_O upon decomposition. This phenomenon can be attributed to the mixed acid pre‐oxidation process, where concentrated sulfuric acid induces dehydration of coal molecules, leading to the formation of ether bonds and anhydrides, both of which do not decompose into H_2_O.

The in situ mass spectrometric profiles of R‐C, AO‐C, and OH‐C resemble their respective DTG curves, further illustrating that OFGs indeed influence the pyrolysis behavior of coal. Among these, the H_2_O generated during the pyrolysis of R‐C and AO‐C primarily originates from the dehydration of ‐OH groups. Compared to R‐C, AO‐C produces a greater amount of H_2_O, and the dehydration peak (at 319 °C) is also at a higher temperature than that of R‐C (306 °C). This is mainly because AO‐C has the highest content of ‐OH groups, and extensive ‐OH dehydration and cross‐linking enhance the thermal stability of coal molecules. However, as can be observed from Figure 2d, this reaction in AO‐C weakens at 319 °C and ceases by 500 °C, indicating that the reaction is largely completed during the melting and rearrangement stage of coal molecules, primarily occurring before rearrangement (<350 °C), thereby significantly reducing its impact on the rearrangement stage. Furthermore, H_2_O is detected between 200–500 °C, indicating that the intermolecular rearrangements of both R‐C and AO‐C occur within this temperature range.

Compared to AO‐C, OH‐C exhibits a broader temperature range for H_2_O release during pyrolysis. This is primarily attributed to the presence of both ‐OH groups and carboxyl groups in OH‐C, which can undergo esterification reactions, and can also engage in dehydration and condensation reactions by themselves. At lower temperatures, the release of H_2_O is mainly associated with ‐OH groups, similar to the cases of R‐C and AO‐C. At higher temperatures, the release of H_2_O is directly related to the carboxyl groups. Generally, long‐chain or aromatic carboxylic acids are more stable than short‐chain carboxylic acids. Since coal is mainly composed of polycyclic aromatic hydrocarbons, the majority of the carboxylic acids obtained after alkali‐oxygen oxidation treatment are aromatic. As shown in Figure 2d, the dehydration peak of OH‐C appears at 412 °C, which coincides with the melting and rearrangement stage. More importantly, the dehydration reaction continues until ≈600 °C. This phenomenon verifies that OH‐C is rich in aromatic carboxylic acids, which broadens the temperature range of cross‐linking reactions, leading to continuous intramolecular rearrangement of coal molecules. Consequently, the coal molecules enter the solidification stage without sufficient time for intermolecular rearrangement.

Figure 2e,f present the 3D TG‐FTIR spectra of R‐C and OH‐C, respectively. The results clearly demonstrate that R‐C releases a complex mixture of volatile components, with decomposition products detected at relatively low temperatures. In contrast, OH‐C exhibits significantly enhanced thermal stability, and no aliphatic compounds are observed in its evolved gases. This finding confirms that the proposed alkali‐oxygen oxidation method effectively removes light organic components, including free aliphatic compounds from coal. Figure 2g–i shows the FTIR spectra of pyrolysis gases from different precursors at 250, 450, and 600 °C. At 250 °C, only the H‐C sample detected CH_4_ and aliphatic compounds. This is mainly due to the strong oxidizing nature of the mixed acid, which disrupts the molecular structure of coal, leading to the cleavage of long‐chain aliphatic hydrocarbons and the formation of volatile short‐chain aliphatic compounds. Additionally, this process increases the number of active edge sites, thereby promoting the cleavage of side chains and the generation of gases such as CH_4_. It is noteworthy that when the temperature is raised to 450 °C or even 600 °C, CH_4_ and aliphatic compounds were detected in all samples except the OH‐C sample. This further confirms that the alkali‐oxygen oxidation method we proposed can effectively remove the free light components in coal and does not introduce excessive active edge sites into the coal molecular structure, which is crucial for improving the ICE of HC materials. Besides, at 450 °C, the OH‐C sample released the most CO_2_, corresponding to the decarboxylation reaction of its stable aromatic carboxyl groups, consistent with previously reported in situ H_2_O mass spectrometry analysis results.

Figure S2 (Supporting Information) presents the scanning electron microscope (SEM) images of the synthesized coal‐based HC samples. All samples exhibit a micrometer‐sized blocky structure. Higher magnification images reveal that the surfaces of R‐HC and AO‐HC samples are adorned with smaller particles, which may be attributed to impurities within the hard carbon. In contrast, such particles are not observed on the surfaces of H‐HC and OH‐HC samples. Furthermore, the HC particles derived from the pre‐oxidation process exhibit a reduced size, particularly for H‐HC with the vigorous reaction, which severely disrupts the coal structure, leading to particle pulverization.

To further investigate the influence of different pre‐oxidation methods on the microstructure of HC, we employed HRTEM. As shown in Figure S3a (Supporting Information), the HRTEM image of sample R‐HC reveals that the HC derived directly from coal exhibits a dense and well‐ordered graphitic microcrystalline structure with thick carbon layers, resulting in an interlayer spacing of only 0.362 nm, due to the lower content of OFGs, which leads to a smaller steric hindrance during the melting rearrangement process. In contrast, the interlayer spacing of sample AO‐HC increases from 0.362 to 0.375 nm (Figure S3b, Supporting Information). However, AO‐HC still displays long‐range ordered lattice fringes with fewer closed pores, which is attributed to the fact that air pre‐oxidation mainly introduces ‐OH functional groups into the coal, and these ‐OH groups begin to dehydrate and condense before the melting rearrangement, which is not effective in inhibiting the rearrangement of graphite microcrystals. This observation is consistent with the results of in situ thermogravimetric analysis coupled with infrared spectroscopy.

The H‐HC sample exhibits a short‐range disordered microcrystalline structure (Figure S3c, Supporting Information), which is due to the vigorous mixed acid oxidation reaction that shears the coal molecules, causing the fragmented coal macromolecules to be difficult to polymerize during the carbonization process. On the other hand, the interlayer spacing of sample OH‐HC expands to 0.377 nm, exhibiting a turbostratic microcrystalline structure with abundant closed pores (Figure S3d, Supporting Information). This microcrystalline structure is more favorable for the transport and storage of Na^+^ ions. The development of this advantageous microcrystalline structure can be attributed to its abundant OFGs, which create effective steric hindrance during molecular rearrangement. Notably, the presence of thermally stable aromatic carboxylic acids significantly reduces intermolecular condensation efficiency during high‐temperature carbonization, thereby inhibiting sufficient time for complete intermolecular rearrangement.

**Figure 3:**
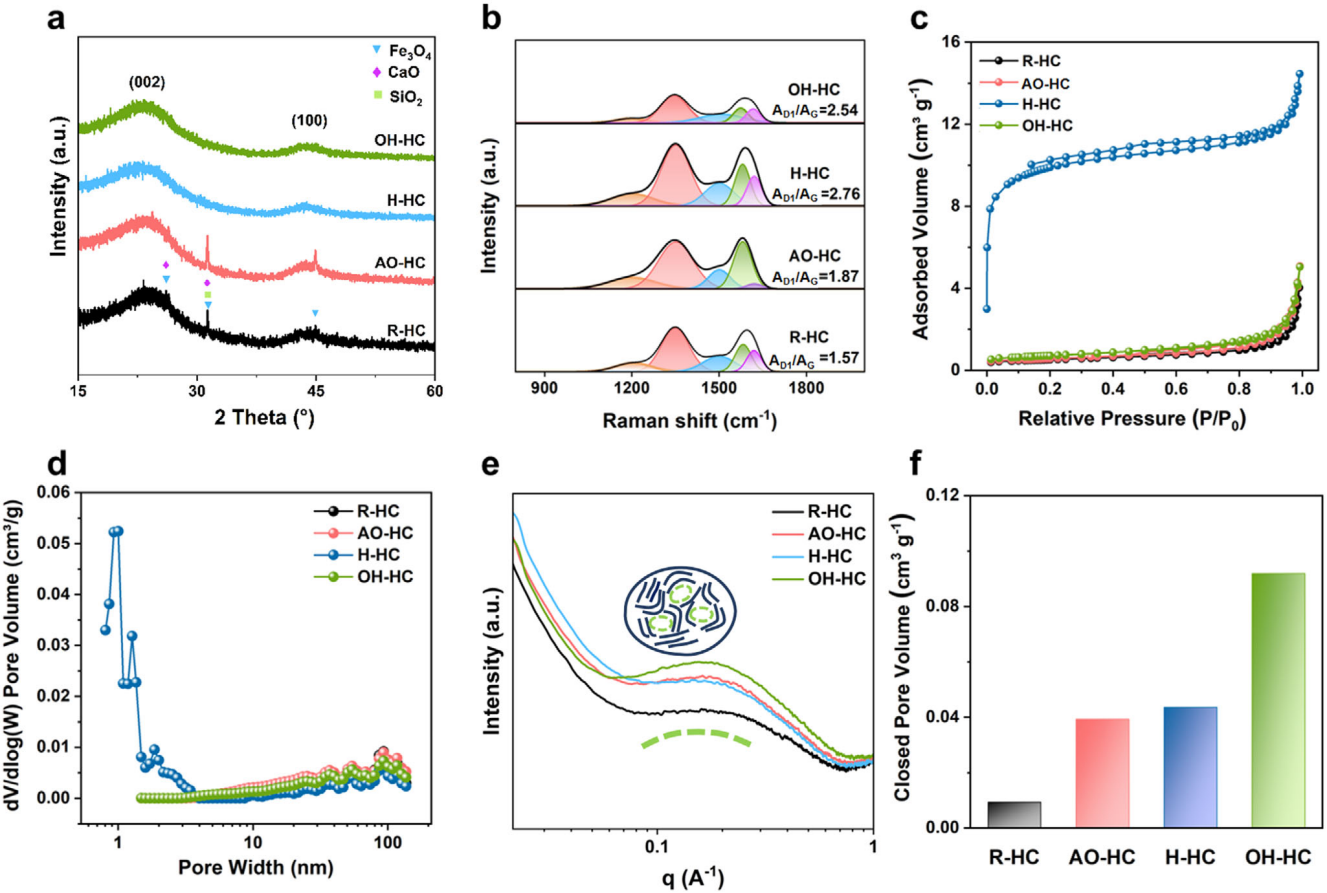
a) XRD patterns, b) Fitted Raman spectra, c) the N_2_ adsorption‐desorption isotherms, d) and their corresponding pore‐size distributions, e) SAXS patterns, and f) closed pore volumes of R‐C, AO‐C, H‐C, and OH‐C.

The microcrystalline structure of coal‐based HC was further analyzed by X‐ray diffractometry (XRD) and Raman spectra. As shown in **Figure** **3**a, peaks of SiO_2_, Fe_3_O_4,_ and CaO were observed in the XRD spectra of the R‐HC and AO‐HC. Notably, no distinct impurity peaks were observed in the XRD patterns of H‐HC and OH‐HC, indicating that these two pre‐oxidation methods effectively remove ash from the coal. While acid treatment is commonly employed for ash removal in reported studies, our proposed alkali‐oxygen oxidation method, as shown in Table S2 (Supporting Information), also achieves efficient de‐ashing, significantly reducing the ash content from 1.61 to 0.26 wt.%. The effective removal of ash is beneficial for both the cycling stability and rate performance of the hard carbon. All samples have two amorphous diffraction peaks at 23° and 43°, representing the (002) and (100) crystallographic planes, respectively.^[^ ^31^ ^]^ Compared to R‐HC, the (002) peaks of AO‐HC, H‐HC, and OH‐HC are shifted to the left, indicating the expansion of their interlayer spacing. In typical coal‐derived carbon materials, disordered carbon layers and graphitic layers coexist. This coexistence allows the (002) diffraction peak to be deconvoluted into two sub‐peaks (Figure S4, Supporting Information): one centered at ≈20°, corresponding to defective disordered carbon structures (pseudo‐graphitic), and the other at ≈24°, attributed to the graphitic domains (graphitic‐like). The ratio of pseudo‐graphitic subpeak areas in R‐HC is only 11.11% with a highly disordered microcrystalline structure, indicating that a significant melting rearrangement occurred during the carbonization process. The ratio of pseudo‐graphitic subpeak areas increases to 21.34% in OH‐HC, demonstrating that carboxyl groups in coal can reduce intermolecular condensation efficiency during high‐temperature carbonization.

Differential Scanning Calorimetry (DSC) curves of R‐C and OH‐C samples are very different (Figure S5, Supporting Information). The DSC curve of sample R‐HC shows a distinct endothermic peak at 479 °C, corresponding to the melting process associated with the transition from a solid crystalline to a liquid amorphous state. A strong exothermic peak is observed ≈558 °C, which is primarily attributed to the polycondensation of polycyclic aromatic hydrocarbons and the formation of long‐range ordered graphene layers. In contrast, no significant endothermic or exothermic peaks are detected in the DSC curve of sample HO‐HC. This result further confirms that the introduction of carboxyl groups can effectively inhibit the melting and rearrangement reactions of coal molecules. The calculated interlayer spacing (d_002_) based on the Bragg formula (Equation S1, Supporting Information) of all the samples is shown in Table S3 (Supporting Information). The d_002_ of R‐HC, AO‐HC, H‐HC, and OH‐HC are 0.368, 0.375, 0.381, and 0.379 nm, respectively, which basically coincide well with the HRTEM results.

In addition, the lateral size (La) and stacking height (Lc) of all samples were calculated using the Scherrer formula (Equation S2, Supporting Information) and summarized in Table S3 (Supporting Information). La represents the average width of graphite crystals along the a‐axis, while Lc represents the average stacking height along the c‐axis. The values of La and Lc are indicative of changes in the microcrystalline structure of the hard carbon.^[^ ^32^ ^]^ Compared to R‐HC (La = 2.494 nm, Lc = 1.757 nm), both La and Lc were reduced for all pre‐oxidized coal‐based HC samples, indicating a decrease in the degree of graphitization. Notably, H‐HC exhibited the smallest La (1.991 nm), attributed to the excessive cleavage of coal molecules by the mixed acid. OH‐HC exhibited the smallest Lc (1.605 nm), primarily due to the high content of carboxyl and ‐C‐O‐C groups in the precursor, which cross‐link during carbonization. This cross‐linking hinders the stacking of microcrystals, resulting in the formation of thinner graphite‐like layer structures.

Raman Spectra of R‐HC, AO‐HC, H‐HC, and OH‐HC are shown in Figure S6 (Supporting Information), which can be deconvoluted into five peaks (Figure 3b), corresponding to the D_1_, D_2_, D_3_, D_4_, and G bands, located at 1350, 1620, 1500, 1200, and 1580 cm^−1^, respectively.^[^ ^33^ ^]^ The D_1_ band represents disordered or defective graphitic domains, the D_2_ band represents amorphous carbon, the D_3_ band represents few‐layer graphene, the D_4_ band represents sp^2^‐sp^3^ hybridized structures or C‐C/C═C stretching vibrations, and the G band represents the sp^2^‐hybridized graphitic layer structure. Typically, the ratio of the integrated areas (A_D1_/A_G_) is used to indicate the degree of carbon structural order.^[^ ^34^ ^]^ The calculated A_D1_/A_G_ values for samples R‐HC, AO‐HC, H‐HC, and OH‐HC are summarized in Table S3 (Supporting Information), with values of 1.57, 1.87, 2.76, and 2.54, respectively. The R‐HC sample exhibited the smallest A_D1_/A_G_ ratio, indicating a more ordered graphitic structure. In contrast, the pre‐oxidation treatments increased the A_D1_/A_G_ ratios for all samples, demonstrating that the pre‐oxidation strategy effectively prevents the graphitization of graphitic microcrystals. Notably, the H‐HC sample exhibited the highest A_D1_/A_G_ ratio, indicating a more disordered graphitic microcrystal structure, which is consistent with the XRD results.

To systematically investigate the effect of different pre‐oxidation methods on the pore structure of coal‐based hard carbon, we conducted N_2_ adsorption‐desorption tests on all samples. As shown in Figure 3c, the adsorption‐desorption curves of R‐HC, AO‐HC, and OH‐HC samples highly overlap, with specific surface areas of 1.84, 1.94, and 2.56 m^2^ g^−1^, respectively. These results indicate that air pre‐oxidation and alkali‐oxygen oxidation treatments have a limited impact on the open‐pore structure of coal‐based HC. In contrast, mixed‐acid pre‐oxidation significantly disrupts the molecular structure of coal, inducing the formation of abundant open pores and increasing the specific surface area of H‐HC samples to 37.12 m^2^ g^−1^. Notably, this high specific surface area may adversely affect the ICE of the material. Further analysis of the pore size distribution in Figure 3d reveals that R‐HC, AO‐HC, and OH‐HC samples exhibit highly consistent pore size distribution characteristics, further confirming the lack of open‐pore structures in these samples. In contrast, H‐HC samples display a pore structure predominantly composed of micropores, mainly attributed to the deep etching effect of mixed acid on the coal matrix.

To comprehensively characterize the internal pore structure of coal‐based HC, we employed a multi‐scale analysis combining small‐angle X‐ray scattering (SAXS) and true density measurements. The SAXS spectra (Figure 3e) show that all samples display a distinct shoulder peak ≈0.1 Å^−1^, which originates from nanopore scattering.^[^ ^35^ ^]^ Notably, the shoulder peak for R‐HC (without pre‐oxidation) is relatively weak, whereas OH‐HC exhibits the most pronounced shoulder peak, indicating a significantly higher population of nanopores in OH‐HC compared to the other samples. Based on Archimedes’ principle, we used helium as the analytical carrier gas to determine the true density of the samples and calculated the closed‐pore volume using Eq. S3 (Supporting Information) (Figure 3f), showing that the OH‐HC sample possesses the highest closed‐pore volume (0.0919 cm^3^ g^−1^), which is 9.8 times, 2.3 times, and 2.1 times higher than that of R‐HC (0.00934 cm^3^ g^−1^), AO‐HC (0.0393 cm^3^ g^−1^), and H‐HC (0.0436 cm^3^ g^−1^), respectively. The combined results of SAXS and true density measurements confirm that the proposed alkali‐oxygen oxidation method can significantly enhance the closed‐pore content of coal‐based HC materials, which is mainly attributed to the fact that this method not only effectively dissolves the organic light components embedded in the coal structure, creating pores within the coal, but also introduces carboxyl functional groups into the coal molecules. These carboxyl groups can continuously cross‐link and decompose to release gases over a wide temperature range, further contributing to the formation of internal closed pores.

**Figure 4:**
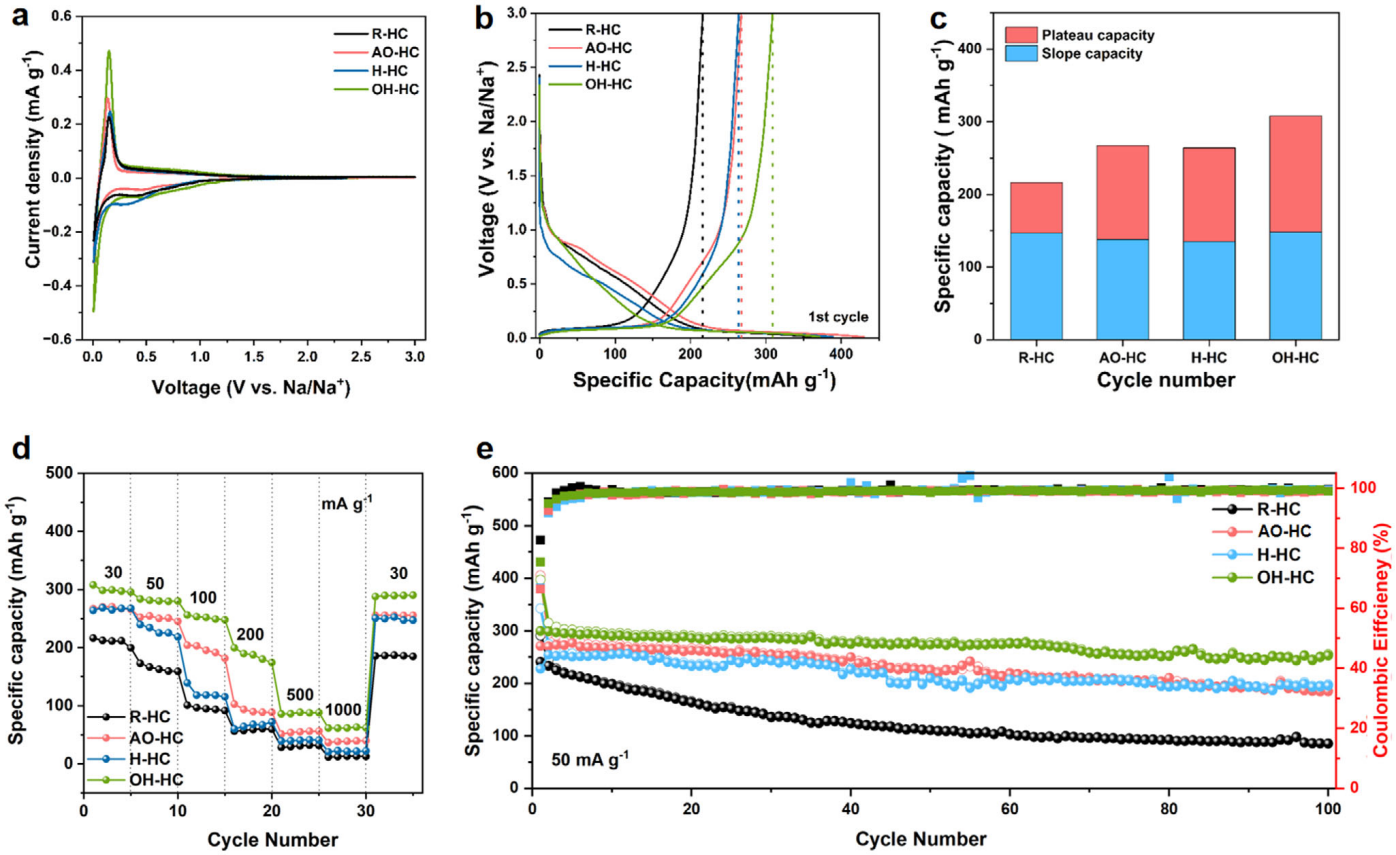
a) The first cycle of CV curves at 0.01 mV s^−1^, b) the initial discharge‐charge curves at the current density of 30 mA g^−1^, c) specific capacities from the plateau region (<0.10 V) and sloping region (>0.10 V) contributions, d) rate performance, and e) cycling performance at 50 mAh g^−1^ of R‐HC, AO‐HC, H‐HC, and OH‐HC.

To evaluate the electrochemical performance of all samples, we assembled half‐cells using metallic sodium as the counter electrode. **Figure** **4**a shows the first‐cycle cyclic voltammetry (CV) curves of R‐HC, AO‐HC, H‐HC, and OH‐HC electrodes at a scan rate of 0.01 mV s^−1^. All electrodes exhibit a broad cathodic peak at ≈0.5 V, which is primarily attributed to the decomposition of the electrolyte and the formation of the solid electrolyte interphase (SEI) layer.^[^ ^36^ ^]^ Additionally, a pair of sharp redox peaks is observed between 0.1 and 0.01 V, corresponding to the intercalation/deintercalation process of Na^+^ ions within the carbon layers and the sodium deposition/stripping in closed pores. The peak areas below 0.1 V for the R‐HC, AO‐HC, H‐HC, and OH‐HC electrodes increase sequentially, suggesting a gradual increase in their plateau capacities. Figure S7 (Supporting Information) displays the first three CV cycles of all electrodes. Compared to the other electrodes, the OH‐HC electrode shows the best overlap in the last two cycles of the CV curves and exhibits the highest peak current (Figure S7d, Supporting Information), indicating its good reversibility. In contrast, the H‐HC electrode exhibits the largest irreversible peak (Figure S7c, Supporting Information), which is mainly due to its larger specific surface area and more surface defects. This leads to the decomposition of the electrolyte on its surface, forming a thicker SEI layer and causing irreversible adsorption of sodium ions.

Figure 4b displays the initial charge/discharge profiles of all electrodes at a current density of 30 mA g^−1^. The discharge curves of all samples consist of a low‐voltage plateau region (<0.1 V) and a high‐voltage slope region (>0.1 V).^[^ ^37^ ^]^ Compared to the R‐HC electrode (216 mAh g^−1^), the pre‐oxidized AO‐HC, H‐HC, and OH‐HC electrodes demonstrate significantly improved reversible capacities of 267, 264, and 308 mAh g^−1^, respectively, with their ICE remarkably increasing from 59.6% to 66.8%, 64.0%, and 80.1%. This performance enhancement primarily originates from the OFGs introduced by pre‐oxidation, which effectively suppress the melting rearrangement during graphitization, thereby expanding the interlayer spacing and providing more active sites for sodium‐ion storage.

Notably, the OH‐HC electrode exhibits the most outstanding electrochemical performance among all samples, which can be attributed to the dual advantages of the alkali‐oxygen oxidation method: i) it effectively extracts the light organic components (including some oxygen‐containing compounds) from the coal structure, preventing detrimental chemical interactions between these free molecules and the carbon framework; ii) the bonded structures formed during this process, if left untreated, would decompose during subsequent carbonization and generate excessive defect sites, significantly compromising the ICE of electrodes. Furthermore, the superior reversible capacity of the OH‐HC electrode is also closely associated with its optimal interlayer spacing and larger closed‐pore volume. The capacity contributions of the sloping and plateau regions for all electrodes are shown in Figure 4c, and their source data are based on the initial‐cycle charge curve shown in Figure S8 (Supporting Information). As shown in Figure S9 (Supporting Information), the galvanostatic charge curves from the 1st to the 30th cycle correspond to the source data of the R‐HC and OH‐HC electrodes presented in Figure 4c. These curves reveal that the capacity contributions of the sloping and plateau regions vary with cycling due to gradual capacity loss and increasing electrochemical polarization. For a fair comparison of the initial electrochemical behavior, we therefore analyzed only the initial‐cycle charge curves of each electrode to evaluate their respective capacity contributions in different voltage regions. Notably, while the R‐HC electrode exhibits significant polarization (Figure S9a, Supporting Information), the charge curves of OH‐HC remain highly consistent across cycles (Figure S9b, Supporting Information), demonstrating its superior electrochemical reversibility. The sloping capacities of the R‐HC (147 mAh g^−1^) and OH‐HC (148 mAh g^−1^) electrodes remained largely unchanged, while their plateau capacities increased significantly from 69 to 160 mAh g^−1^. This enhancement can be attributed to the optimized interlayer spacing and the presence of abundant closed pores, which facilitate efficient ion transport and provide additional storage sites for Na^+^ ions.

Figure 4d comparatively evaluates the rate capabilities of R‐HC, AO‐HC, H‐HC, and OH‐HC electrodes within the current density range of 0.03–1 A g^−1^. The electrochemical measurements demonstrate that all pre‐oxidized electrodes (AO‐HC, H‐HC, and OH‐HC) exhibit superior rate performance compared to the pristine R‐HC electrode. Remarkably, the OH‐HC electrode delivers outstanding reversible capacities of 308, 256, 200, and 85 mAh g^−1^ at current densities of 30, 100, 200, and 500 mA g^−1^, respectively, significantly surpassing those of untreated R‐HC (216.5, 109, 56.3, and 28.3 mAh g^−1^). This exceptional rate capability can be ascribed to the expanded interlayer spacing and abundant closed‐pore structure in OH‐HC, which collectively facilitate rapid electron transport and ion diffusion while providing additional active sites for sodium storage. In contrast, the AO‐HC and H‐HC electrodes exhibit relatively inferior rate performance, due to the presence of thermally unstable OFGs on their surfaces that fail to effectively inhibit structural rearrangement and their restricted closed‐pore volume. The cycling stability tests (Figure 4e) reveal that at 50 mA g^−1^, the R‐HC electrode suffers from severe capacity degradation, decreasing from an initial 194 mAh g^−1^ to merely 92 mAh g^−1^ after 100 cycles, corresponding to a poor capacity retention of 47.4%. In striking contrast, the pre‐oxidized samples show significantly enhanced cycling stability, of which the OH‐HC electrode exhibits the most impressive performance (maintaining 85% of its initial capacity after 100 cycles). Comprehensive comparison with literature data confirms that the OH‐HC electrode outperforms other reported coal‐based hard carbons in both rate capability and sodium storage capacity (Table S4, Supporting Information).

**Figure 5:**
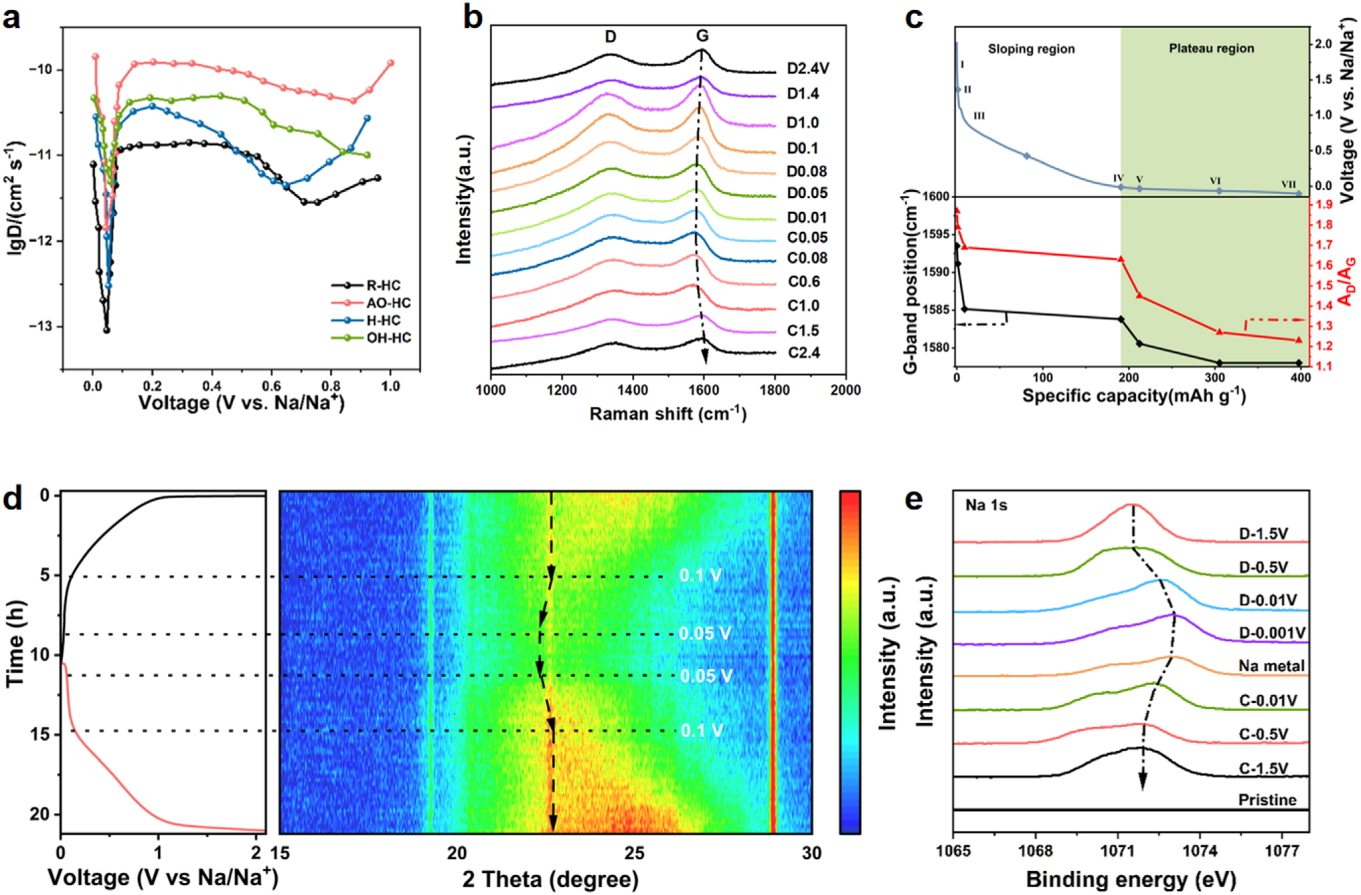
a) The calculated sodium ion chemical diffusion coefficients of R‐HC, AO‐HC, H‐HC, and OH‐HC electrodes during the discharge process, b) the operando Raman spectra, c) the evolution of the G‐band position and A_D_/A_G_ as a function of discharge capacity, d) the operando XRD patterns during the discharge‐charge process, and e) ex situ XPS at different cut‐off voltages of OH‐HC electrodes.

To further investigate the storage kinetics of coal‐based HC, the initial charging and discharging processes of all samples were tested with the galvanostatic intermittent titration technique (GITT) at a pulsed current of 20 mA g^−1^ in Figure S10 (Supporting Information). The diffusion coefficients of Na^+^ (*D*_Na_^+^) were calculated according to Fick's second law (Eq. S4, Supporting Information):^[^ ^38^ ^]^ the diffusion coefficients of Na^+^ at the corresponding voltages for all samples during the discharge process are shown in **Figure** **5**a. During the sodiation process at voltages above 0.1 V, all electrodes exhibit relatively high sodium ion diffusion coefficients (*D*_Na_^+^), which can be primarily attributed to the favorable reaction kinetics resulting from sodium ion adsorption at defect sites and intercalation into graphite layers with an enlarged interlayer spacing (d > 0.37 nm). As the depth of discharge increases, the *D*_Na_^+^ values demonstrate a characteristic trend of initial decrease followed by an increase within the voltage range of 0.10–0.01 V.^[^ ^39^ ^]^ The sharp decline in *D*_Na_^+^ observed at the 0.05 V plateau region is likely associated with the sluggish diffusion kinetics of sodium ions into the graphite layers or closed pores. Notably, at 0.05 V, R‐HC shows an exceptionally low *D*_Na_^+^ of 9.1 × 10^−14^ cm^2^ s^−1^, significantly lower than that of pre‐oxidized coal‐based HC materials. In contrast, OH‐HC achieves a remarkably high *D*_Na_^+^ of 5×10^−12^ cm^2^ s^−1^ substantially exceeding those of AO‐HC (1.5 × 10^−12^ cm^2^ s^−1^) and H‐HC (3 × 10^−13^ cm^2^ s^−1^). FTIR analysis was performed on all samples. As illustrated in Figure S11 (Supporting Information), compared with R‐HC, the hard carbon materials obtained after pre‐oxidation treatment all exhibit introduced OFGs on their surfaces. In particular, C═O groups are successfully incorporated through different pre‐oxidation methods. These groups are considered active sites for sodium adsorption, where the reaction C═O + Na^+^ → C‐O‐Na occurs. This process corresponds to a surface‐controlled pseudocapacitive mechanism with extremely fast kinetics.^[^ ^40^ ^]^ Consequently, AO‐HC, H‐HC, and OH‐HC all demonstrate higher *D*_Na_^+^ than R‐HC above 0.1 V. Notably, AO‐HC, which shows the highest C═O peak intensity, exhibits the largest *D*_Na_^+^ in this potential region. The relatively lower *D*_Na_^+^ above 0.1 V for H‐HC can be attributed to its higher concentration of ‐OH groups, which strongly adsorb Na^+^ ions via hydrogen bonding and electrostatic interactions, hindering desorption and increasing diffusion resistance. Importantly, the OH‐HC sample contains epoxy ether groups, which provide weakly polar adsorption sites and reduce the energy barrier for Na^+^ desolvation.^[^ ^41^ ^]^ Coupled with its optimized interlayer spacing and abundant closed pore structure, these features synergistically enhance Na^+^ ion diffusion, resulting in the largest *D*_Na_^+^ below 0.1 V. Figure S12 (Supporting Information) presents the magnified discharge curves of all the electrodes with normalized discharge capacities. The average voltages of the OH‐HC and AO‐HC electrodes are higher than that of the H‐HC electrode, primarily due to the abundant microporous structure in H‐HC, which is consistent with the results of the BET analysis. Besides, the R‐HC electrode exhibits a relatively lower average voltage, which can be attributed to its narrow graphite interlayer spacing that hinders efficient Na^+^ ion intercalation. In contrast, the higher average voltages of the OH‐HC and AO‐HC electrodes mainly result from their highly efficient Na^+^ ion intercalation process, which contributes substantially to their capacity. Notably, the OH‐HC electrode demonstrates the highest average voltage, indicating that its intercalation process provides the highest capacity contribution.

The structural evolution and sodium storage mechanism of the OH‐HC material during electrochemical cycling were systematically investigated using operando Raman spectroscopy (Figure 5b), which shows that within the discharge range from 2.4 V (open circuit voltage, OCP) to 0.01 V, the D‐band peak position of the material remains stable (≈1336 cm^−1^), but its relative intensity shows a continuous decreasing trend. This spectral characteristic change is mainly attributed to the preferential adsorption of Na^+^ ions at defect sites and on the surface of pores in the carbon material, which significantly inhibits the breathing vibration mode of the carbon ring edge structures. Notably, the G‐band characteristic peak exhibits a three‐stage red‐shift phenomenon: initially, during the discharge process, it shifts from 1593.5 cm^−1^ (OCP) to 1583.8 cm^−1^ (0.1 V), corresponding to the adsorption process of sodium ions at defect sites and between carbon layers with a large interlayer spacing (d > 0.40 nm); subsequently, a larger shift occurs in the range of 0.1 to 0.05 V (to 1578.0 cm^−1^), indicating the intercalation of sodium ions within the graphitic‐like layers. Furthermore, in the range of 0.05 to 0.01 V, the position of the G‐band peak remains essentially unchanged, indicating that sodium is mainly stored through pore‐filling behavior.^[^ ^42^ ^]^ The fitted Raman spectra and the corresponding peak areas of the D and G bands at different voltages are provided in Figure S13 (Supporting Information). According to the fitting results, the A_D_/A_G_ values at voltages of 2.4, 1.4, 1.0, 0.1, 0.08, 0.05, and 0.01 V are 1.87, 1.79, 1.69, 1.63, 1.45, 1.27, and 1.23, respectively. The variation of the A_D_/A_G_ ratio further confirms this mechanism: in the sloping voltage region (2.4–0.1 V), the ratio decreases from 1.87 to 1.63, reflecting the adsorption of sodium ions at defect sites; while in the plateau region (0.1–0.001 V), it drops sharply to 1.23, confirming the dominant role of the pore‐filling mechanism. The above evidence demonstrates that the sodium storage behavior of this material involves both interlayer intercalation and pore‐filling mechanisms (Figure 5c).

To further elucidate the electrochemical reaction mechanism of the OH‐HC electrode during the charge and discharge processes, the operando XRD characterization of the electrode was conducted during cycling, as shown in Figure 5d. The intensity of the (002) peak increases gradually, as indicated by the color transition from blue to red. From the charging process (represented by the red curve on the left), it can be observed that within the voltage range of 0.01–0.05 V, the peak position of the (002) plane (yellow) remains largely unchanged.^[^ ^43^ ^]^ This suggests that sodium storage within the closed pores does not affect the interlayer spacing of the carbon framework. Between 0.05 and 0.1 V, the (002) peak broadens and shifts slightly toward higher angles, corresponding to the extraction of Na^+^ ions from the interlayer spaces.^[^ ^44^ ^]^ In the voltage range from ≈0.1 to 2 V, the (002) peak continues to broaden, while the position of the maximum intensity (deepest red) remains stable. This behavior is attributed to the “pseudo‐desorption” of Na^+^ among highly disordered carbon structures. The change in the (002) peak of its discharge process is basically opposite, indicating that this battery has excellent electrochemical reversibility.^[^ ^45^ ^]^

Furthermore, ex situ XPS analysis was employed to further confirm the chemical state of sodium in OH‐HC under different sodiation states. As illustrated in Figure 5e, no characteristic sodium signal was detected in the pristine OH‐HC electrode. Upon discharging to 1.5 V, a distinct Na 1s peak emerged at 1071.6 eV, confirming the presence of Na⁺ ions. Further discharge to 0.5 V resulted in peak broadening, while the peak position remained largely unchanged, indicating that sodium remained in the ionic form adsorbed at surface defect sites.^[^ ^46^ ^]^ When discharged to 0.01 V, the Na 1s peak shifted to a higher binding energy, and further shifted upon reaching 0.001 V, aligning with the peak position of metallic sodium, suggesting the presence of sodium in a quasi‐metallic state at this potential.^[^ ^47^ ^]^ Upon recharging to 1.5 V, the binding energy gradually decreased and reversibly returned to 1071.7 eV, demonstrating the reversible transformation of sodium from the quasi‐metallic state back to the ionic state. All the above in situ and ex situ analyses indicate that the Na^+^ ion storage mechanism in OH‐HC is based on a hybrid “adsorption‐intercalation/filling” model.

## 3 Conclusion

In summary, various OFGs are introduced into coal molecules through different pre‐oxidation methods. Notably, our proposed alkali‐oxygen oxidation method not only facilitates the dissolution of organic light components, thereby avoiding the introduction of defects and creating pores within the coal, but also successfully incorporates carboxyl functional groups into the coal molecules. In situ TG‐IR‐MS analysis reveals that carboxyl groups play a pivotal role in prolonging the relaxation time of coal molecules. This effect elevates the temperature window for intramolecular carbon rearrangement from 500 up to 600 °C. As a result, insufficient intermolecular rearrangement occurs prior to solidification, ultimately leading to an expanded interlayer spacing and an increased closed pore volume in the resulting coal‐derived HC. The optimized OH‐HC exhibits a significantly enhanced capacity of 308 mAh g^−1^ with an ICE of 80.1%. Furthermore, in situ and ex situ characterization confirm the presence of an “adsorption‐intercalation/filling” hybrid mode during different sodium storage stages. This study investigates the surface chemistry of coal‐based HC and the manipulation of OFGs to prevent intramolecular rearrangement during pyrolysis, thereby improving its sodium storage performance and practical application.

## 4 Experimental Section

### Materials Preparation

Coal sourced from Hei Shan, Xinjiang, China, served as the precursor for the synthesis of hard carbon (HC). The raw coal was subjected to ball milling and subsequently crushed to pass through a 200‐mesh sieve, resulting in the sample labeled as R‐C. Then, 25 mL of 30% H_2_O_2_ and 1 M NaOH solution were placed in a flask, to which 1 g of raw coal was added. The mixture was oxidized at 70 °C in a water bath for 7 h, then dried to obtain the product, which was washed multiple times with hydrochloric acid and deionized water until neutral, named as OH‐C. For comparison, the raw coal was placed in a muffle furnace and oxidized at 300 °C with a heating rate of 5 °C min^−1^ for 2 h, labeled as AO‐C. In a 160 mL mixed acid solution (H_2_SO_4_ and HNO_3_ in a volume ratio of 3:1), 10 g of raw coal was added and stirred in an ice‐water bath for 8 h, then washed with water until neutral, labeled as H‐C. Subsequently, R‐C, AO‐C, H‐C, and OH‐C were placed in a tubular furnace under an argon atmosphere and calcined at 1300 °C to obtain the hard carbons, designated as R‐HC, AO‐HC, H‐HC, and OH‐HC, respectively.

### Materials Characterization

The crystallographic phases of the samples were identified through X‐ray diffraction (XRD, Bruker D8) with Cu‐Kα radiation (λ = 1.54 Å) and Raman spectroscope (Bruker Senterra R200‐L). The specific surface area and pore structure were evaluated using the Brunauer‐Emmett‐Teller (BET, ASAP 2460) method, which involved N_2_ adsorption/desorption measurements (BELSORPmax). Additionally, the small‐angle X‐ray scattering (SAXS) technique was employed to further analyze the pore structures. Thermogravimetric analysis (TG) was conducted using a state‐of‐the‐art synchronous thermal analyzer (NETZSCHSTA449F3, Germany). Fourier transform infrared (FTIR) spectroscopy was carried out with a high‐precision FTIR spectrometer (VERTEX 70 RAMI). X‐ray photoelectron spectroscopy (XPS) data were acquired on an ESCALab 250 Xi system (Thermo Fisher Scientific), utilizing Al Kα radiation for analysis. The morphological and microstructural characteristics of the samples were examined using a scanning electron microscope (SEM, Hitachi SU8010) and a high‐resolution transmission electron microscope (HRTEM, FEI Tecnai F30).

### Electrochemical Tests

The working electrode was prepared by mixing the active material, Super‐P, and polyvinylidene fluoride (PVDF) binder in a mass ratio of 85:5:10. N‐methyl‐2‐pyrrolidone (NMP) was added to form a homogeneous slurry, which was then coated onto a copper foil current collector. The electrode was dried in a vacuum oven at 80 °C for 12 h, after which it was cut into small discs with a diameter of 12 mm to obtain the half‐cell anode. All electrochemical test coin cells (CR2025) were assembled in an argon‐filled glove box. The half‐cell consisted of the coal‐based HC material as the anode, metallic sodium as the counter electrode, a glass fiber GF/F separator, and an electrolyte of 1.0 M NaClO_4_ dissolved in a solvent mixture of EC:PC:DEC (1:1:1 by volume). The tests included galvanostatic charge‐discharge (GCD), rate performance, and galvanostatic intermittent titration technique (GITT), all conducted using a Land battery testing system (CT2001A, Land, China). Cyclic voltammetry (CV) was performed on an electrochemical workstation (CHI660E).

## Conflict of Interest

The authors declare no conflict of interest.

## Supporting information



Supporting Information

## Data Availability

The data that support the findings of this study are available from the corresponding author upon reasonable request.
