# Supplementary material for: Manipulating Surface Chemistry on the Microarchitecture of Coal‐Based Hard Carbon for Improved Sodium Storage
Source: Adv Sci (Weinh). 2025 Sep 23;12(46):e13835. doi: 10.1002/advs.202513835 (PMC12697760; doi:10.1002/advs.202513835)
Supplement: Supplementary file 1 — Supporting Information [file ADVS-12-e13835-s001.docx]

Supporting Information

Manipulating Surface Chemistry on the Microarchitecture of Coal-Based Hard Carbon for Improved Sodium Storage

Wenhai Zhang, Ruizhen Song, Hong Meng, Yakun Tang^*^, Yue Zhang^*^, Lang Liu^*^, Ping Han, Limin Deng, Yuliang Cao^*^

**Formulas**

1.Bragg formula (Eq.(S1)) : $d_{002}(nm)=\frac{n\lambda}{2sin\theta}$

where λ is the wavelength (0.15406 nm) of the X-ray, θ is the position of (002) peaks.

2.Scherrer formulae (Eq. (S2)) : $L(nm)=\frac{k\lambda}{\beta cos\theta}$

where β is the full width at half maximum (FWHM) of peaks, θ is the position of peaks, k is equal to 1.84 and 0.9 when calculating La and Lc, respectively.

3.Closed pore volume calculation formula (Eq. (S3)) :

$$\rho_{\mathrm{ske}}g \mathrm{cm}^{-3}=\frac{\frac{z}{N_{A}\mathrm{mol}^{-1}}*M}{({a cm)}^{2}*\sin\left( 60^{\circ} \right)*b cm}$$

Z=4；M=12 g mol^-1^；a=2.46 Å；b=2*d*_002_

$$\frac{1}{\rho_{\mathrm{ture}}g \mathrm{cm}^{-3}}=(\frac{1}{\rho_{\mathrm{ske}}g \mathrm{cm}^{-3}}+V_{\mathrm{cp}}g \mathrm{cm}^{-3})$$

4.Fick's second law (Eq.(S4)): $D_{\mathrm{Na}^{+}}=\frac{4}{\pi\tau}{(\frac{m_{c}V_{c}}{M_{c}S})}^{2}({\frac{\Delta E_{s}}{\Delta E_{t}})}^{2}$

in which Vc is the molar volume of sample, mc is the mass, Mc is the molar mass, S is the surface area of the electrode disc, τ is the duration of the pulse, ΔEt is the difference between two continuous open circuit potentials (V), ΔES is the voltage offset (V) when a current pulse is applied.

**Figures**
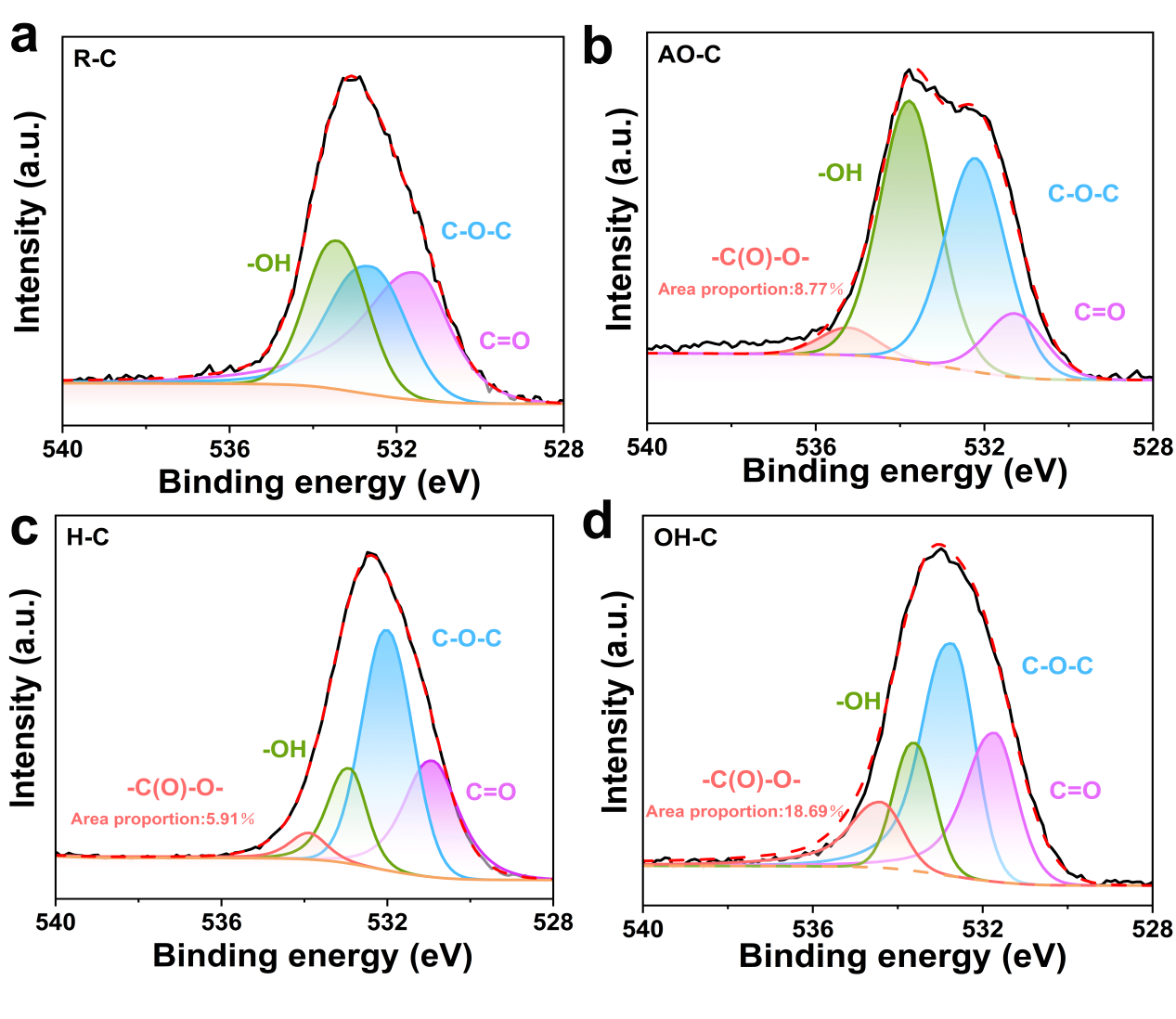


Figure S1. The high-resolution O 1s XPS spectra of R-C (a), A-C (b), H-C (c), and OH-C (d).


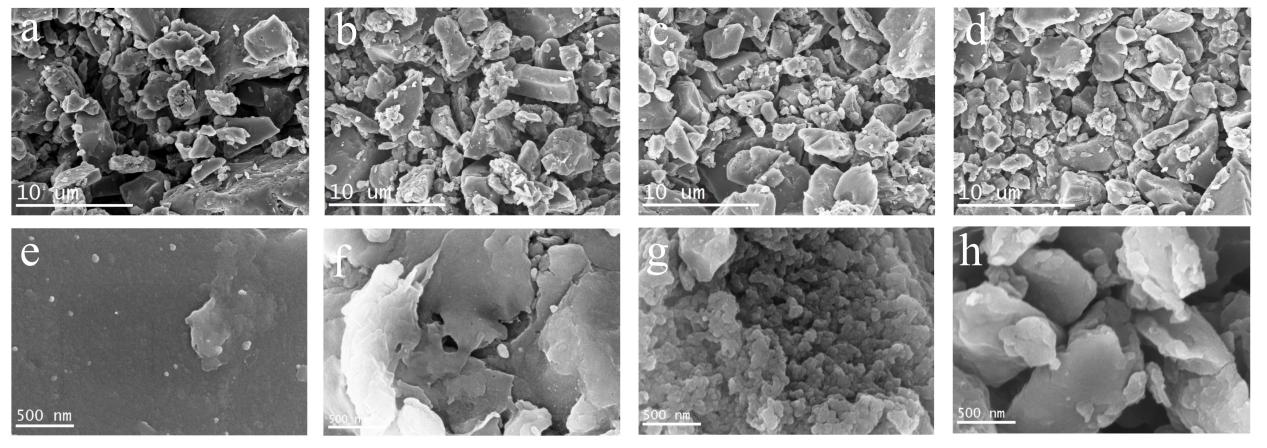


Figure S2. SEM images of R-HC (a, e), AO-HC (b, f), H-HC (c, g) and OH-HC (d, h)


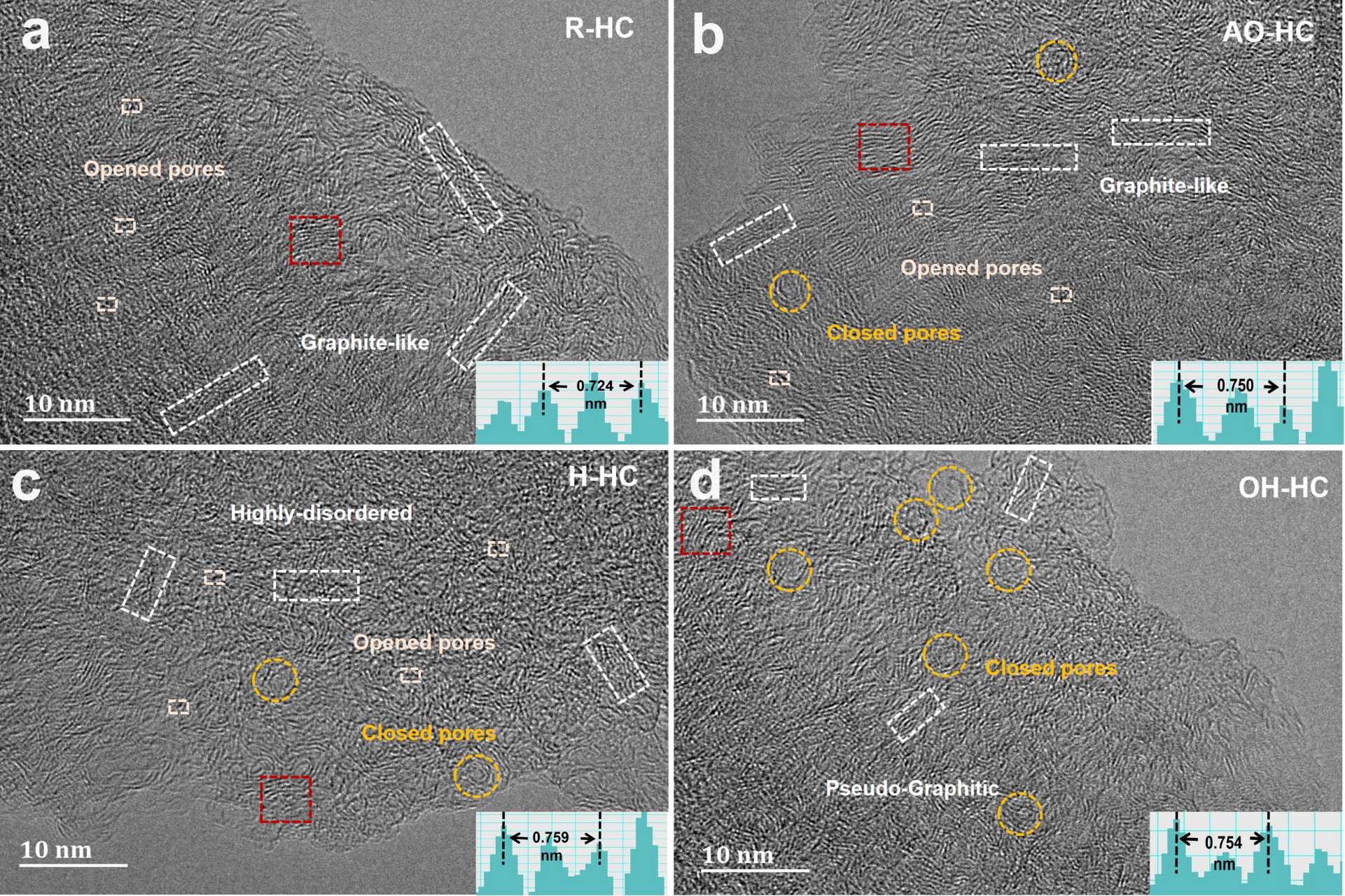


Figure S3. The HRTEM images of (a) R-HC, (b) AO-HC, (c) H-HC, (d) OH-HC.


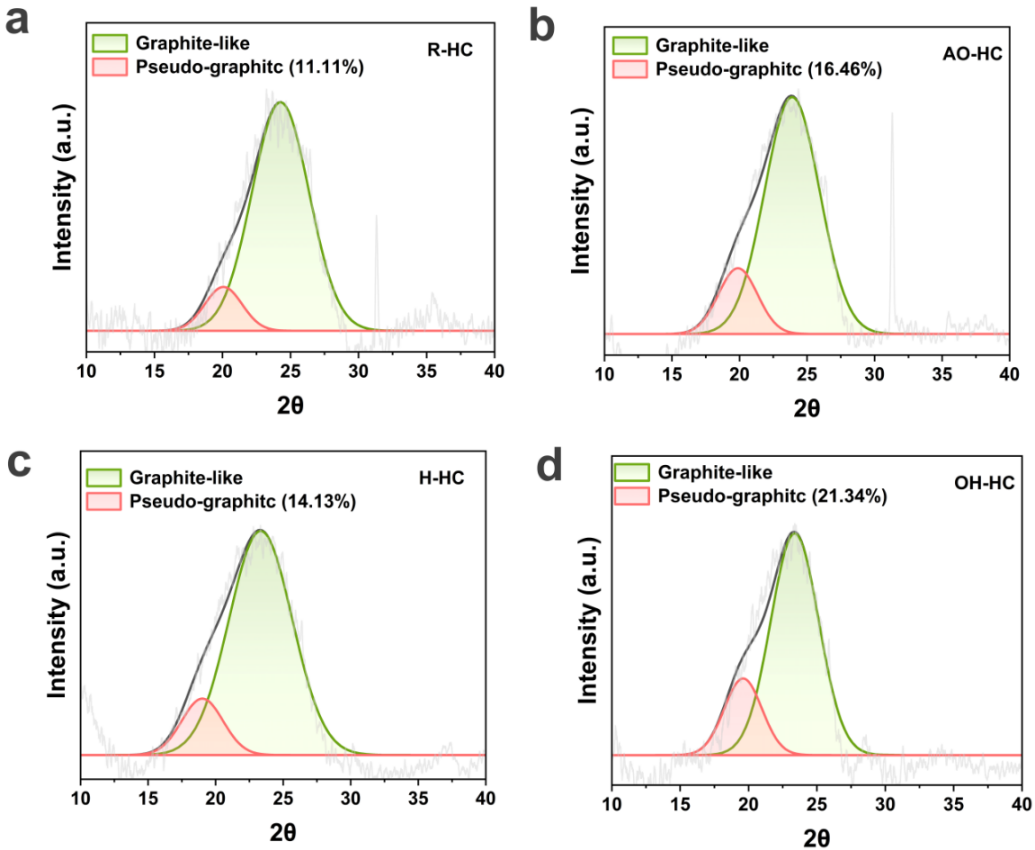


Figure S4. Fitted XRD patterns of R-C, AO-C, H-C, and OH-C samples.


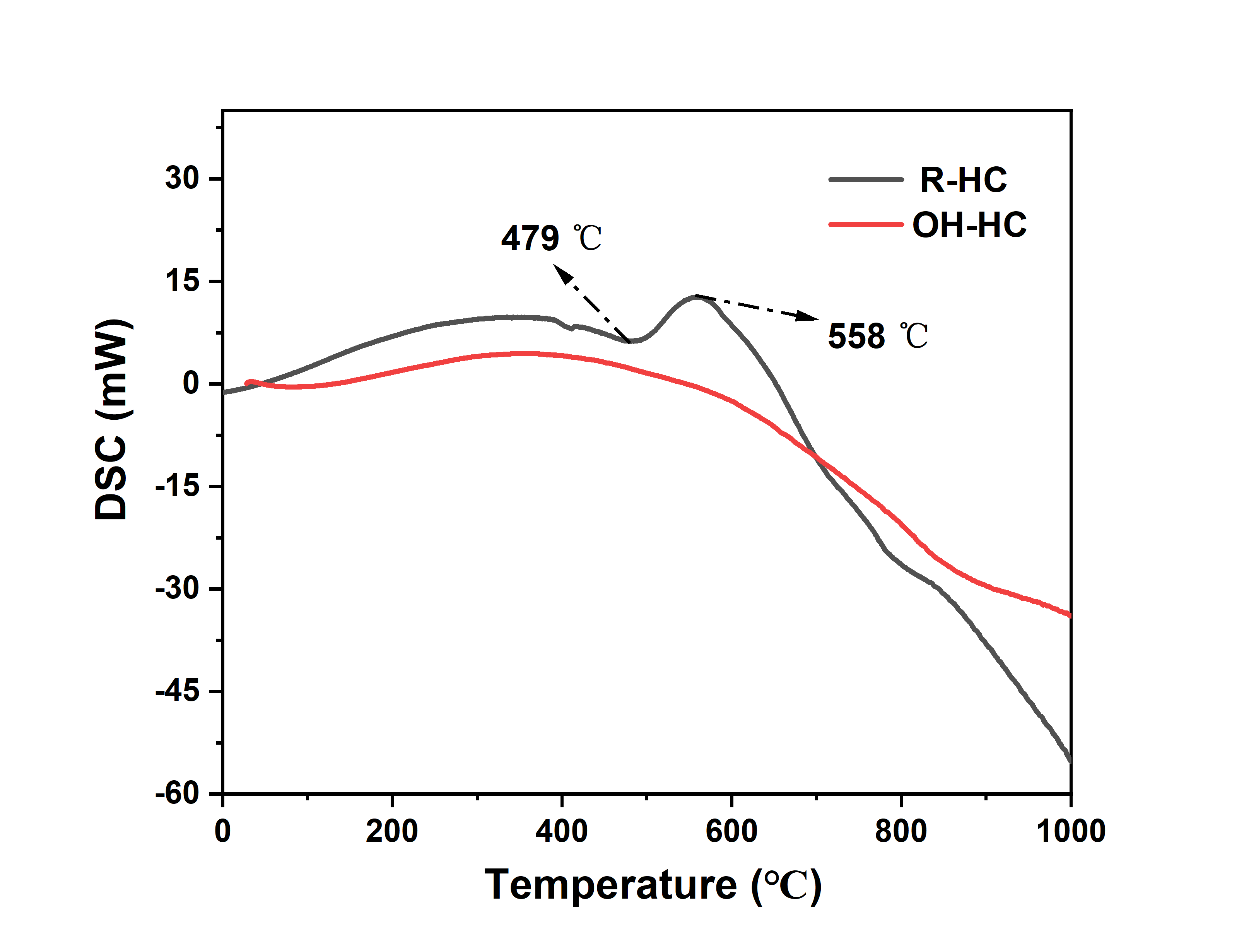


Figure S5. DSC curves of R-C, and OH-C samples.

Figure S6. Raman Spectra of R-HC, AO-HC, H-HC and OH-HC.


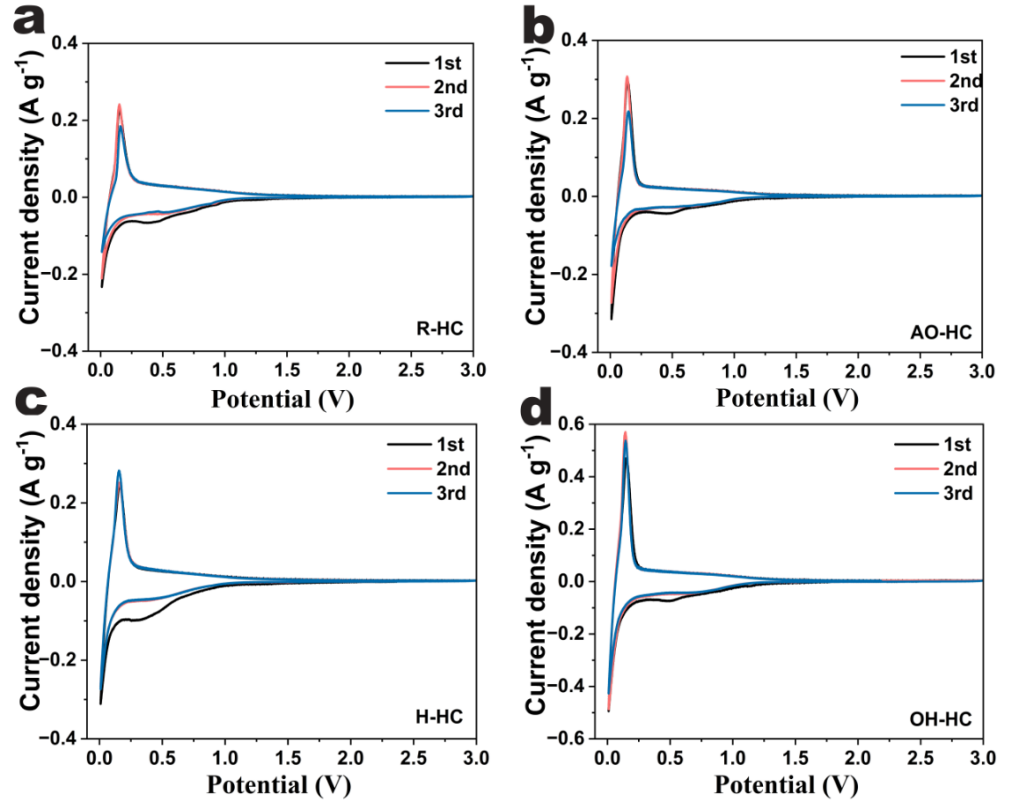


Figure S7. CV curves for the first three cycles of R-HC (a), AO-HC (b), H-HC (c), and OH-HC (d).


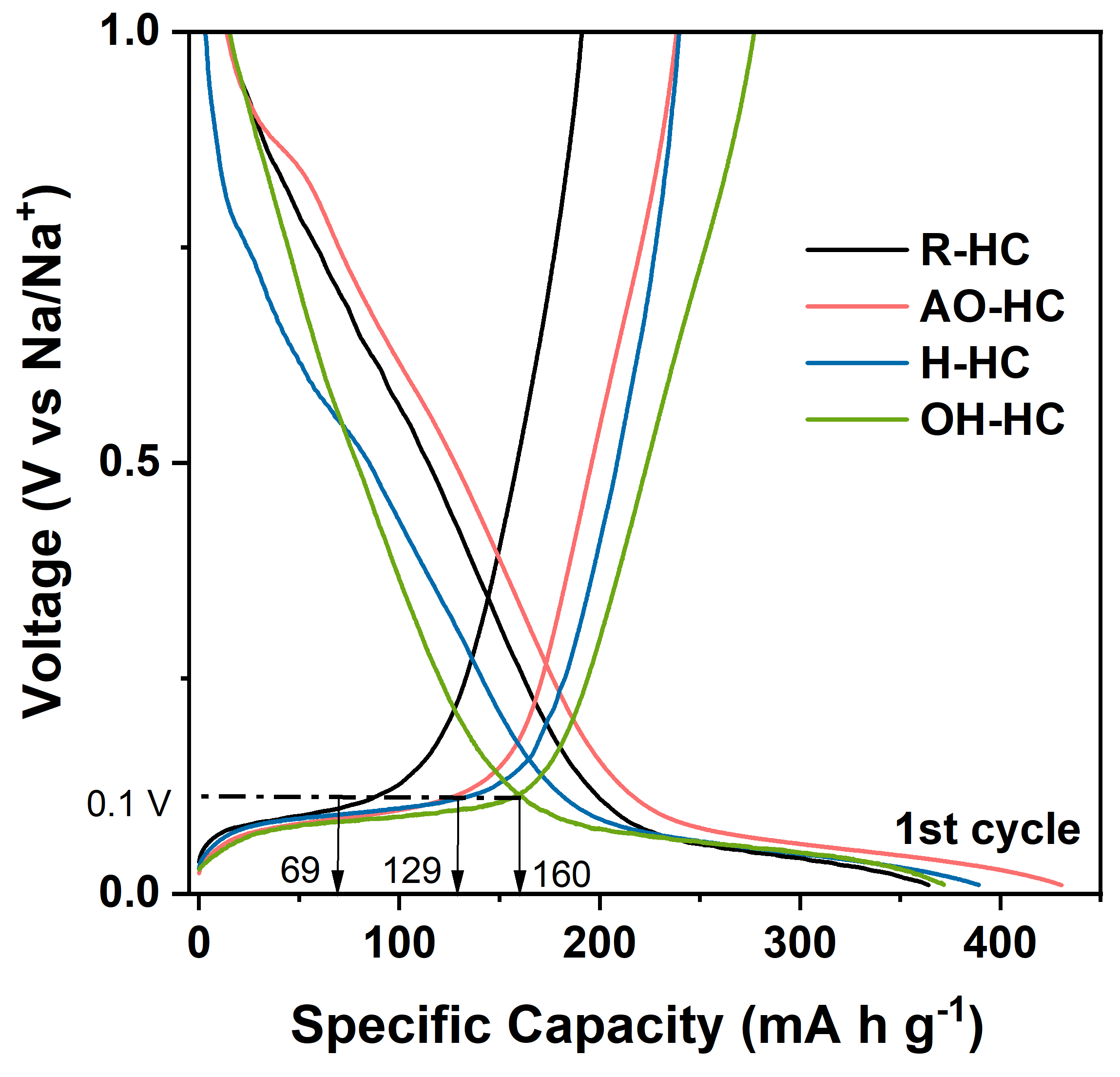


Figure S8. The enlarged initial discharge-charge curves within the voltage range of 0.01-1.0 V.


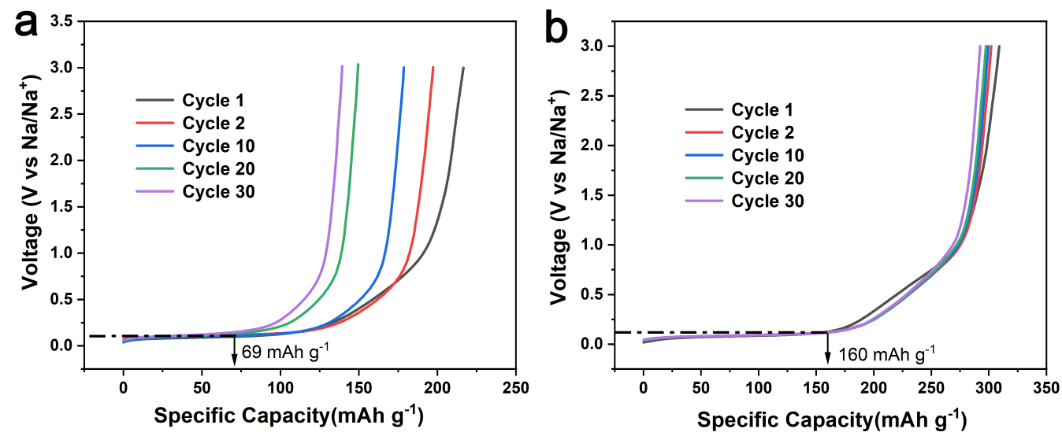


Figure S9. The galvanostatic charge curves from cycle 1 to cycle 30 of R-HC (a) and OH-HC (b) electrodes.

Figure S10. Galvanostatic intermittent titration technique (GITT) potential profiles of R-HC, AO-HC, H-HC, and OH-HC electrodes during the first discharge cycle.


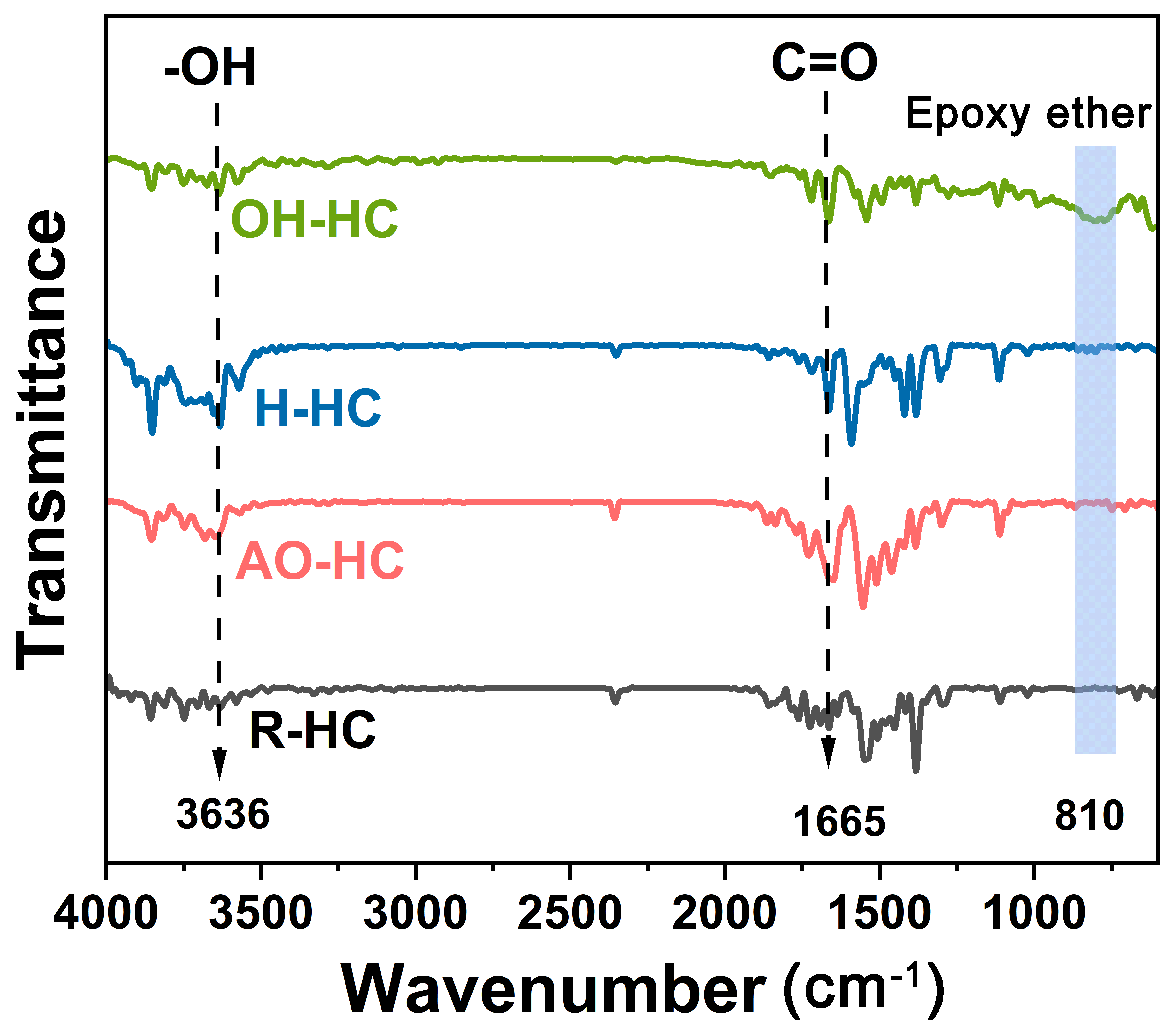


Figure S11. FTIR spectra of R-HC, AO-HC, H-HC, and OH-HC.


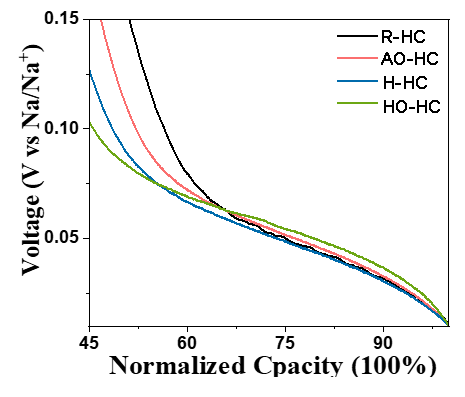


Figure S12. The magnified discharge curves of the R-HC, AO-HC, H-HC, and OH-HC electrodes with normalized discharge capacities.


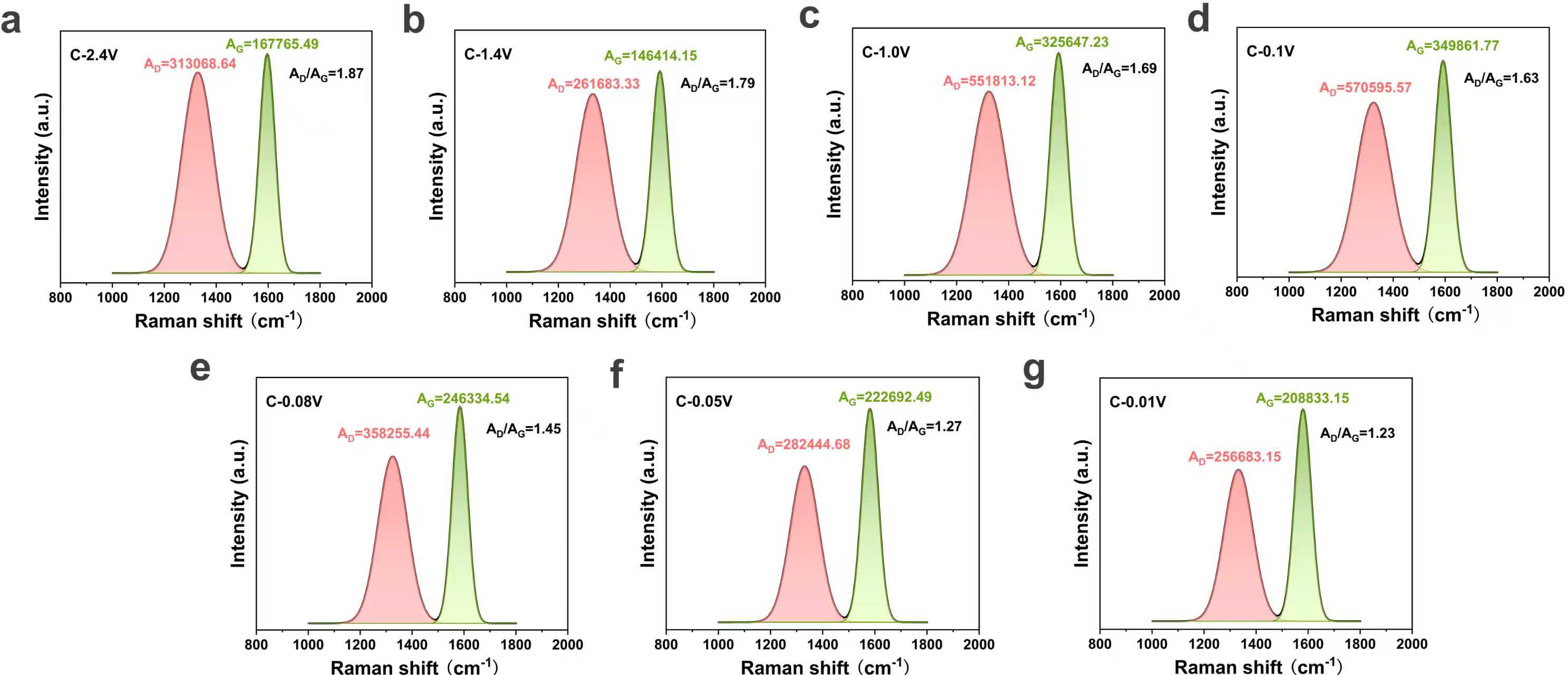


Figure S13. The fitted Raman spectra and the corresponding peak areas of the D and G bands at different voltage.

**Tables**

Table S1. Percentages of C, O and O1s from XPS results of the R-C、AO-C、H-C and OH-C.

| Samples | C  (at%) | O  (at%) | C=O (at%) | 1. O-C   (at%) | -OH  (at%) | -O-C=O (at%) |
| --- | --- | --- | --- | --- | --- | --- |
| R-C | 88.43 | 11.57 | 39.62 | 32.8 | 27.55 | - |
| AO-C | 82.38 | 17.62 | 10.66 | 37.37 | 43.20 | 8.77 |
| H-C | 71.06 | 27.83 | 28.61 | 49.49 | 16.00 | 5.91 |
| OH-C | 84.73 | 15.27 | 22.51 | 43.97 | 14.83 | 18.69 |

Table S2. Proximate analyses (wt%) of bituminous coal precursor and pretreated coal.

| Sample | M_ad_ | A_d_ | V_daf_ | FC_d_ |
| --- | --- | --- | --- | --- |
| R-C | 1.13 | 1.61 | 36.8 | 60.46 |
| OH-C | 1.59 | 0.26 | 35.07 | 64.76 |

M_ad_, moisture; A_d_, ash; V_adf_, volatile matter; FC_d_, fixed carbon.

Table S3. Physical parameters of the obtained material

| Sample | 2θ_002_(°) | d_002_(nm) | La(nm) | 2θ_100_ (°) | Lc(nm) | A_D1_/A_G_ |
| --- | --- | --- | --- | --- | --- | --- |
| R-HC | 24.17 | 0.368 | 2.494 | 43.69 | 1.757 | 1.57 |
| AO-HC | 23.69 | 0.375 | 2.089 | 44.13 | 1.754 | 1.87 |
| H-HC | 23.28 | 0.381 | 1.991 | 43.92 | 1.730 | 2.76 |
| OH-HC | 23.41 | 0.379 | 2.054 | 43.87 | 1.605 | 2.54 |

Table S4. A survey of electrochemical performances of coal baesd hard carbon electrode materials reported in the open literature.

| **Material** | **Specific capacity** | **Rate capacity** | **Reference** |
| --- | --- | --- | --- |
| Anthracite | 222 mAh g^-1^ at  30 mA g^-1^ | 190 mAh g^-1^ at  150 mA g^-1^ | [1] |
| Subbituminous Coal | 291 mAh g^-1^ at  20 mA g^-1^ | 95 mAh g^-1^ at  200 mA g^-1^ | [2] |
| Bituminous coal | 274.2 mAh g^-1^ at  30 mA g^-1^ | 172.9 mAh g^-1^ at  200 mA g^-1^ | [3] |
| Anthracite | 308 mAh g^-1^ at  30 mA g^-1^ | 150 mAh g^-1^ at  300 mA g^-1^ | [4] |
| Lignite coal and Sucrose | 356 mAh g^-1^ at  30 mA g^-1^ | 207 mAh g^-1^ at  200 mA g^-1^ | [5] |
| Subbituminous coal | 284.4 mAh g^-1^ at  20 mA g^-1^ | 151.8 mAh g^-1^ at  200 mA g^-1^ | [6] |
| Anthracite | 282 mAh g^-1^ at  50 mA g^-1^ | 205 mAh g^-1^ at  200 mA g^-1^ | [7] |
| Bituminous coal | 306 mAh g^-1^ at  30 mA g^-1^ | 218 mAh g^-1^ at  200 mA g^-1^ | [8] |
| bituminous coal | 326 mAh g^-1^ at  20 mA g^-1^ | -  - | [9] |
| Subbituminous coal | 308 mAh g^-1^ at  30 mA g^-1^ | 200 mAh g^-1^ at  200 mA g^-1^ | This work |

Reference:

1、Y. Li, Y. S. Hu, X. Qi, X. Rong, H. Li, X. Huang, and L. Chen, “Advanced Sodium-Ion Batteries Using Superior Low Cost Pyrolyzed Anthracite Anode: towards Practical Applications,” Energy Storage Materials (2016): 191-197, <http://10.1016/j.ensm.2016.07.006>

2、H. Lu, S. Sun, L. Xiao, J. Qian, X. Ai, H. Yang, A.-H. Lu, and Y. Cao, “High-Capacity Hard Carbon Pyrolyzed from Subbituminous Coal as Anode for Sodium-Ion Batteries,” ACS Applied Energy Materials (2019): 729-735, <http://10.1021/acsaem.8b01784>

3、Z. Lou, H. Wang, D. Wu, F. Sun, J. Gao, X. Lai, and G. Zhao, “Microcrystalline Regulation of Bituminous Coal Derived Hard Carbon by Pre-Oxidation Strategy for Improved Sodium-Ion Storage,” Fuel (2022): 122072, <http://https://doi.org/10.1016/j.fuel.2021.122072>

4、K. Wang, F. Sun, H. Wang, D. Wu, Y. Chao, J. Gao, and G. Zhao, “Altering Thermal Transformation Pathway to Create Closed Pores in Coal-Derived Hard Carbon and Boosting of Na^+^ Plateau Storage for High-Performance Sodium-Ion Battery and Sodium-Ion Capacitor,” Advanced Functional Materials (2022): 2203725, <http://https://doi.org/10.1002/adfm.202203725>

5、H. Chen, N. Sun, Q. Zhu, R.A. Soomro, and B. Xu, “Microcrystalline Hybridization Enhanced Coal-Based Carbon Anode for Advanced Sodium-Ion Batteries,” Advanced Science (2022): 2200023, <http://https://doi.org/10.1002/advs.202200023>

6、W. Song, Y. Tang, J. Liu, S. Xiao, Y. Zhang, Y. Gao, C. Yang, and L. Liu, “Mild Pretreatment Synthesis of Coal-Based Phosphorus-Doped Hard Carbon with Extended Plateau Capacity as Anodes for Sodium-Ion Batteries,” Journal of Alloys and Compounds (2023): 169384, <http://https://doi.org/10.1016/j.jallcom.2023.169384>

7、R. Li, B. Yang, A. Hu, B. Zhou, M. Liu, L. Yang, Z. Yan, Y. Fan, Y. Pan, J. Chen, T. Li, K. Li, J. Liu, and J. Long, “Heteroatom Screening and Microcrystal Regulation of Coal-Derived Hard Carbon Promises High-Performance Sodium-Ion Batteries,” Carbon (2023): 118489, <http://https://doi.org/10.1016/j.carbon.2023.118489>

8、M. Y. Su, K. Y. Zhang, E. H. Ang, X. L. Zhang, Y. N. Liu, J. L. Yang, Z. Y. Gu, F. A. Butt, and X. L. Wu, “Structural Regulation of Coal-Derived Hard Carbon Anode for Sodium-Ion Batteries via Pre-Oxidation,” Rare Metals (2024): 2585-2596, <http://10.1007/s12598-023-02607-3>

9、G. Liu, J. Yuan, Z. Li, H. Li, C. Wang, Z. Zeng, C. Hu, J. Yang, B. Yuan, J. Zhang, and Z. Wu, “Structure Reconstruction Strategy for Controlled Pore Closure via Surface Coating in Coal-Based Hard Carbon for Enhancing Sodium Storage Performance,” Carbon (2025): 120085, <http://https://doi.org/10.1016/j.carbon.2025.120085>
